# *Oviductus Ranae* as a Functional Food for Ovary Protection: Ameliorating Premature Ovarian Failure and Modulating PI3K/Akt and Apoptosis Pathways in Rats

**DOI:** 10.1002/fsn3.72089

**Published:** 2026-07-07

**Authors:** Hongyu Zhao, Yu Wang, Xianchun Cheng, Xingyao He, Xueyuan Sun, Lin Di, Zhen Yang

**Affiliations:** ^1^ Jilin Academy of Chinese Medicine Sciences Changchun Jilin P.R. China; ^2^ Key Laboratory of TCM Pharmacology Jilin Academy of Chinese Medicine Sciences Changchun Jilin P.R. China; ^3^ College of Chemistry and Life Sciences Changchun University of Technology Changchun Jilin P.R. China

**Keywords:** Bcl‐2/Bax, estrogen, follicles, ovary, *Oviductus Ranae*, PI3K‐AKT, POF, uterus

## Abstract

Due to the increasing pressure of modern society, premature ovarian failure (POF) has become a widespread issue in women. POF not only impacts women's fertility but also heightens the risk of osteoporosis and cardiovascular diseases. *Oviductus Ranae* (OR) is a natural product that is used as medicine and food to exert “estrogen‐like effects.” Previous studies have reported that the compound improves follicle growth, which may be beneficial for POF. Thus, an experiment was designed to explore OR's positive effects on POF rats. POF rat models were established by intraperitoneal injection of cyclophosphamide (CTX), and OR was orally administered to the rats. Subsequently, serum estrogen and gonadotropin levels were measured by radioimmunoassay. Uterine status, endometrial epithelial thickness, and follicle numbers at different stages in ovaries were analyzed by H&E staining, and follicle atresia by TUNEL staining. Western blot was used to detect the expression levels of key proteins in the phosphatidylinositol 3‐kinase (PI3K)/AKT serine/threonine kinase 1 (Akt) signaling pathway, as well as B‐cell lymphoma 2 (Bcl‐2) and Bcl‐2‐associated X protein (Bax) in ovarian tissues. The results showed that OR administration to POF rats alleviated estrous cycle disorder, increased estrogen levels (*p* < 0.05), decreased gonadotropin levels (*p* < 0.05), mitigated uterine atrophy, increased endometrial epithelial thickness (*p* < 0.01), ovarian weight (*p* < 0.05), secondary and Graafian follicle numbers (*p* < 0.05), while reducing secondary and Graafian follicle atresia rate (*p* < 0.05). These beneficial effects may be attributed to OR‐mediated upregulation of the PI3K/Akt signaling pathway and modulation of the Bcl‐2/Bax apoptotic axis in ovarian tissue. In conclusion, OR can improve the follicular condition in ovaries and relieve symptoms in POF rats.

## Graphical abstract

Schematic illustration of the experimental design for evaluating the protective effects of *Oviductus Ranae* (OR) against cyclophosphamide (CTX)‐induced premature ovarian failure (POF) in rats. Female Wistar rats were intraperitoneally injected with CTX (100 mg/kg on Day 0, followed by 10 mg/kg daily) for 14–16 days to establish the POF model. Rats were then randomly divided into five groups: Control (C), Model (M), Positive drug (P), low‐dose OR (ORL, 200 mg/kg), and high‐dose OR (ORH, 400 mg/kg). The P group received estrogen replacement therapy (estradiol valerate + megestrol acetate) in 5‐day cycles with clomiphene citrate ovulation induction, while OR groups were orally administered OR gel solution for 14–16 consecutive days. Estrous cycle was monitored throughout the treatment period. At the end of the experiment, blood, uterus, and ovaries were collected for serum hormone analysis (E_2_, P, FSH, LH), histopathological examination (H&E and TUNEL staining), and Western blot detection of PI3K/Akt signaling pathway and apoptosis‐related proteins (Bcl‐2/Bax).

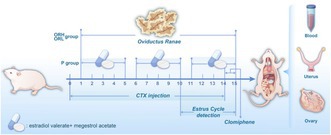

## 1 Introduction

Premature ovarian failure (POF) is a clinical syndrome characterized by menstrual disorders under the age of 40, accompanied by decreased estrogen levels and increased gonadotropin levels. Under normal physiological conditions, menopause typically occurs between the ages of 49 and 52. However, various adverse factors can lead to early menopause, including autoimmune or metabolic disorders, thyroid and adrenal diseases, infection (mumps), genetic factors (chromosomal abnormalities), and iatrogenic factors (chemotherapy) (Ayesha et al. 2016; Wu et al. 2005). According to clinical reports, the incidence of POF in women aged 20, 30, and 40 years is about one in 10,000, one in 1000, and one in 100, respectively (Coulam et al. 1986). With increasing societal pressures, including academic and occupational stress, the prevalence of POF has shown a rising trend in recent years. POF impairs follicular development within the ovaries, preventing the production of mature and viable oocytes, ultimately leading to infertility. This exacerbates concerns regarding declining population growth. Moreover, POF results in reduced estrogen levels in women, which increases the risk of osteoporosis and cardiovascular and cerebrovascular diseases in women. Previous studies have reported a significantly lower incidence of cardiovascular and cerebrovascular diseases in premenopausal women compared to men of the same age. In contrast, the incidence of cardiovascular and cerebrovascular diseases in postmenopausal women is higher than in men of the same age (Reckelhoff 2001; Appelman et al. 2015). An earlier onset of POF is associated with an increased risk of cardiovascular and cerebrovascular diseases in women (Wellons et al. 2012). Currently, the primary clinical treatment for POF is estrogen replacement therapy (ERT) (Smith et al. 2014; Aitken et al. 1973), which improves osteoporosis in women suffering from POF. However, the benefits of ERT on the risk of cardiovascular and cerebrovascular diseases remain questionable (Manson et al. 2003). In addition, ERT increases the risk of breast, ovarian, and uterine cancer, leading to its decreasing use in clinical practice (Rossouw et al. 2013). Therefore, safer and more reliable alternative therapies are required to treat POF.

**FIGURE 1:**
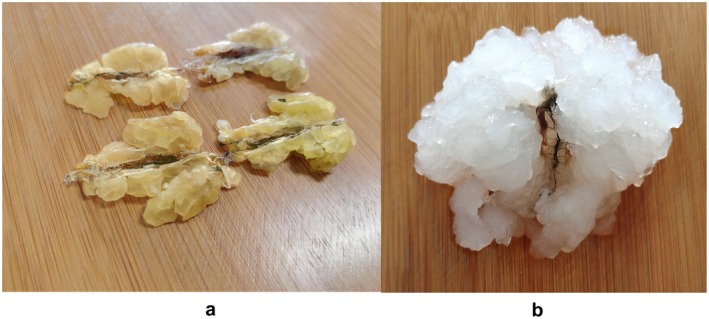
*Oviductus Ranae*: (a) Dried *Oviductus Ranae*; (b) *Oviductus Ranae* after soaking.

*Oviductus Ranae* (OR) is a natural product used both as medicine and food, derived from the dried oviducts of Chinese *Rana chensinensis* (Figure 1). OR contains high proportions of protein, accounting for about 50% of its content. The plant is also rich in steroid hormones, polysaccharides, phospholipids, fatty acids, and 1‐methylhydantoin (Zhang et al. 2019). OR has been consumed for hundreds of years in China, and modern studies have also confirmed its safety (Zhang et al. 2017). Since the Ming Dynasty in China, OR has been used as a nourishing ingredient for women, which was believed to promote female beauty and fertility. Recent studies have reported that OR has an “estrogen‐like effect”. OR can effectively increase serum estrogen levels, thicken the uterine wall and endometrium in mice (Kang et al. 2015), and improve reproductive organ atrophy and osteoporosis secondary to low estrogen levels in rats and mice (Li et al. 2019; Liang et al. 2008). In previous laboratory studies, OR exerted an “estrogen‐like effect” on normal rats, which was negligible in castrated female rats (Zhao et al. 2019). This suggests that its mechanism of action is likely dependent on endogenous ovarian function. Consequently, experiments were conducted to investigate the effects of OR on ovarian function and follicular development. In vitro follicle culture experiments and in vivo rat experiments revealed that the addition of serum containing OR extract promoted follicular growth, reduced follicular atresia, and increased estradiol (E_2_) concentrations in follicular culture medium and serum. Furthermore, the PI3K‐Akt signaling pathway was up‐regulated in both ovaries and follicles after OR treatment (Zhao et al. 2024), which plays a crucial role in ovarian function. Notably, the recruitment of primordial follicles, the proliferation of granulosa cells, luteal formation, and oocyte maturation are all associated with the PI3K‐Akt signaling pathway (Adhikari and Liu 2009). In summary, the “estrogen‐like effect” induced by the follicle‐stimulating effects of OR is hypothesized to benefit pre‐menopausal POF patients by improving ovarian function and increasing estrogen levels.

This study aims to evaluate the health benefits of OR in the context of POF, providing a comprehensive assessment of its effects on ovarian function and estrogen levels. Hence, a POF rat model was established by administering CTX injections. Following the oral administration of OR, various physiological and biochemical parameters were assessed, including estrous cycle duration, serum sex hormone levels, ovarian morphology, follicle count at different developmental stages, follicular atresia rate, and endometrial status. Subsequently, the PI3K‐Akt signaling pathway and key apoptotic proteins Bcl‐2 and Bax in the ovary—both identified in our prior transcriptomics studies—were quantified to systematically analyze the therapeutic efficacy and underlying mechanism of OR in treating POF. This study represents the first systematic investigation into the intervention effect of OR on POF rats and the regulation of its target pathways.

## 2 Materials and Methods

### 2.1 Preparation of OR and Gavage Samples

OR (Figure 1a, Batch No.: 202411A) was provided by Tonghua Dezheng Hall Wild Resources Development Co. Ltd., and was authenticated by Chief Pharmacist Lin Di from Jilin Academy of Chinese Medicine Sciences as the dried oviducts of the female *Rana temporaria chensinensis* David. To prepare the intragastric administration sample, OR was rehydrated using the traditional water‐soaking method. The plant was presoaked in distilled water at a material‐to‐liquid ratio of 1:100 for 16 h, yielding a characteristic gelatinous gel (Figure 1b). The gel was then homogenized using a high‐speed blender to obtain a uniformly dispersed and moderately viscous gel solution, which was subsequently used for intragastric administration in rats.

### 2.2 Animal Source and Group Treatment

This study was reviewed and approved by the Laboratory Animal Ethics Committee of Jilin Academy of Chinese Medical Sciences (Approval No.: JLSZKYDWLL2021‐012) in accordance with the regulations of the Laboratory Animal Ethics Committee. Sixty SPF‐grade female Wistar rats (initial body weight 170–190 g; pre‐experimental body weight 200–220 g) (animal certificate No. SCXK (Liao) 2020‐0001) were purchased from Liaoning Changsheng Biotechnology Co. Ltd. The experimental animals were housed in individually ventilated cages (IVC) cages in the Laboratory Animal Barrier System of Jilin Academy of Chinese Medical Sciences at a temperature of 20°C–25°C, a relative humidity of 55%–70%, and a 12‐h light/dark cycle, with free access to food and water. After 1 week of adaptation, the animals were tested for estrous cycle for 4 consecutive days. A total of 50 female rats with normal estrous cycle and uniform body weight were randomly divided into five groups, including the control group (Group C), model group (Group M), positive group (Group P), low‐dose OR group (Group ORL), and high‐dose OR group (Group ORH), with 10 rats in each group. Except for rats in the control group, the rats in other groups were intraperitoneally injected with CTX (Dalian Melon Biotechnology Co. Ltd., Dalian, China) for 14–16 days. Specifically, 100 mg/kg CTX was injected on the first day, followed by 10 mg/kg daily; the duration of injection was adjusted with the length of gavage, and CTX was not injected on the day of sacrifice. A rat model of ovarian injury was established, while the control group was intraperitoneally injected with an equal volume of normal saline. On the day following model establishment, the low‐ and high‐dose OR groups were intragastrically administered with OR solution at doses of 200 and 400 mg/kg, respectively (Kang et al. 2015; Zhao et al. 2024). The positive group was treated with ERT plus ovulation induction every 5 days as a treatment cycle. Specifically, estradiol valerate solution 0.1 mg/kg was administered intragastrically on Days 1–3; on Day 4, intragastric estradiol valerate (DELPHARM Lille S.A.S., France) solution 0.1 mg/kg was given; on Day 5, megestrol acetate (Qingdao Guohai Biopharmaceutical Co. Ltd., Qingdao, China) solution 0.8 mg/kg was administered, followed by normal saline. In the third treatment cycle, when diestrus was detected, clomiphene citrate (Medochemie Ltd., Cyprus, Cyprus) solution 10 mg/kg was administered daily by gavage (approximately 1–2 doses depending on the length of diestrus in different rats). All intragastric drug doses were calculated based on the recommended human clinical doses converted to equivalent animal doses (Nair and Jacob 2016). The control group and the model group were gavaged with equal volumes of distilled water. Rats in each group were intragastrically administered a volume of 20 mL/kg for 14–16 consecutive days. The rats were sacrificed during the proestrus phase after 14 days of intragastric treatment; the number of days of intragastric administration was slightly adjusted according to the estrus period. Body weights were measured twice every week. In the first proestrus stage after 14 days of gavage, the rats were anesthetized with sodium pentobarbital, blood was collected from the abdominal aorta, and serum was separated for future use. The uterus and both ovaries were excised, weighed, and the organ index was calculated (organ index = organ weight [mg]/body weight [g]). The collected tissues were then used for histopathological examination and molecular analysis.

### 2.3 Estrous Cycle Monitoring

**FIGURE 2:**
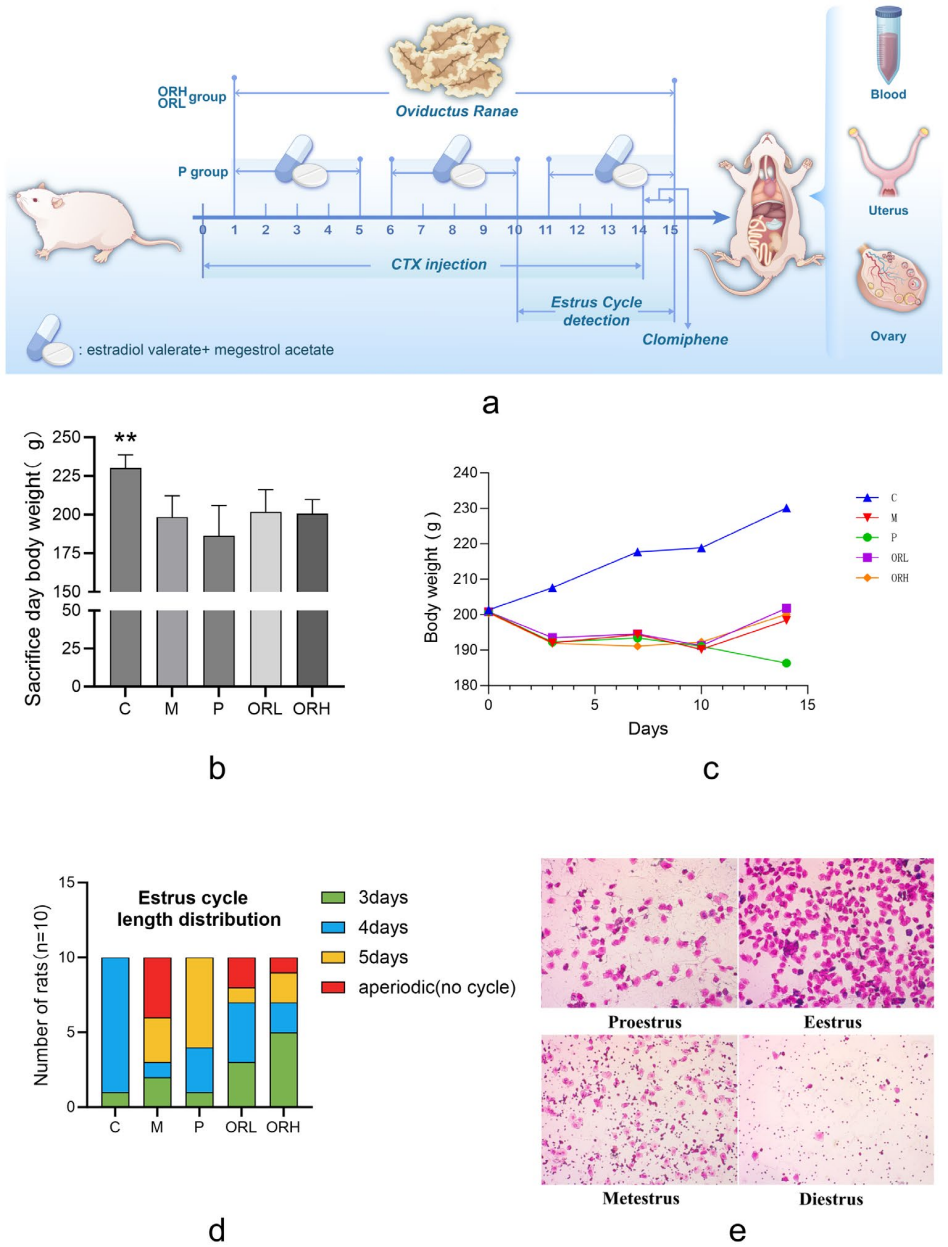
Scheme of animal experiments and changes in body weight and estrous cycle of rats. (a) Scheme of animal experiments. (b) Sacrifice body weight of rats in each group. (c) Changes in body weight of rats in each group from Day 1 to 14 of gavage. (d) Changes in estrous cycle of rats in each group. (e) Vaginal smear staining at different stages of the estrus cycle. C, control group; M, model group; ORH, high‐dose OR group; ORL, low‐dose OR group; P, positive drug group. Data are represented as the mean ± SD from 10 animals in each group. *p < 0.05 and **p < 0.01 versus M group, ^#^ *p* < 0.05 and ^##^ *p* < 0.01 versus P group.

Vaginal secretions were collected by lavage with normal saline and rapid Gram staining was performed to determine the estrous cycle by observing epithelial cell types in secretions under optical microscopy (Ndeingang et al. 2019) (Figure 2e). Female rats exhibiting a regular progression through the proestrus, estrus, metestrus, and diestrus phases over 4–5 consecutive days were classified as having a normal estrous cycle. The duration of the estrous cycle was recorded as the time interval between the first proestrus phase observed after 10 days of drug administration and the subsequent proestrus phase.

### 2.4 Serum Sex Hormone Test

The contents of estradiol (E_2_), progesterone (P), follicle‐stimulating hormone (FSH), and luteinizing hormone (LH) in rat serum were detected by a competitive radioimmunoassay kit (Beijing Northern Institute of Biotechnology Co. Ltd., Beijing, China).

### 2.5 Histological Analysis: Uterus and Ovarian H&E Staining, Ovarian TUNEL Staining, and Follicle Counting

The left ovary and uterus were excised, rinsed with physiological saline, and fixed in 10% formalin for 48 h. The tissues were then subjected to gradient ethanol dehydration for 8 h, followed by xylene dehydration for 4 h, and finally embedded in paraffin for 6 h before sectioning. For uterine histological analysis, 5‐μm sections were prepared from the uterine horn region and stained with hematoxylin and eosin (H&E). Morphological changes in the uterus were observed under a microscope. In each uterine section, three fields of view were randomly selected at 400× magnification, and the thickness of the endometrial epithelial cell layer was measured. The average thickness of these three measurements was recorded as the endometrial epithelial thickness for that sample. For ovarian analysis, 5‐μm sections were obtained from the region with the largest cross sectional area of the ovary. Serial sections were subjected to H&E staining and TUNEL staining, followed by whole‐slide scanning. Ovarian H&E‐stained sections were used to count primary follicles, secondary follicles, Graafian follicles, and corpora lutea. The developmental stages of follicles were classified according to their distinct morphological characteristics: Primary follicles: oocytes surrounded by a single layer of cuboidal or columnar granulosa cells. Secondary follicles: characterized by two or more layers of granulosa cells, with a clearly formed follicular cavity (antrum). Graafian follicles (mature follicles): displayed a large, dilated follicular antrum, with the oocyte and surrounding cumulus cells forming a cumulus‐oocyte complex protruding into the antrum. Corpus luteum: an epithelioid structure composed of numerous luteal cells with abundant cytoplasm, rich in capillaries (Gougeon 1996).

To evaluate follicular atresia, TUNEL assay was performed on ovarian sections. Follicles at different developmental stages (primary, secondary, and Graafian), as identified by parallel H&E‐stained sections, were classified as atretic if they displayed positive TUNEL staining in granulosa cells. The atresia rate for each follicular stage was calculated as the percentage of TUNEL‐positive follicles relative to the total number of follicles at that stage. The data on follicle count and atretic follicle count for each animal were all derived from a single ovarian section, and the visual field area for the counting data was the area of all ovarian tissues in the whole‐slide scan. In this study, primordial follicles were not included in the statistical analysis.

### 2.6 Protein Extraction and Western Blot Analysis

The right ovary of rats was milled at low temperature using a tissue grinder (Ltd—T P‐24 type Gering Scientific Instruments, Beijing, China) and proteins were extracted by centrifugation after low‐temperature incubation with RIPA solution (Shanghai Biyuntian Biotechnology Co. Ltd., Shanghai, China) for 30 min, and the extracted protein concentration was determined using a BCA kit (Shanghai Biyuntian Biotechnology Co. Ltd., Shanghai, China). The extract was separated by SDS‐PAGE gel electrophoresis (Beijing Dingguo Changsheng Biotechnology Co. Ltd., Beijing, China) and transferred to PVDF membrane (Millipore, USA), blocked with TBST buffer containing 5% BSA for 20 min, and 3 mL of the corresponding antibody PI3K (PI3 Kinase p110 delta Rabbit mAb, ABclonal Technology, Wuhan, China), Akt (AKT1 Rabbit mAb, ABclonal Technology, Wuhan, China), phosphorylated Akt (p‐Akt) (Phospho‐Akt‐S473 Rabbit mAb, ABclonal Technology, Wuhan, China), phosphatase and tensin homolog deleted on chromosome 10 (PTEN) (PTEN Bcl‐2 Rabbit mAb, ABclonal Technology, Wuhan, China), Bcl2 (Bcl‐2 Rabbit mAb, ABclonal Technology, Wuhan, China), TBST dilutions (1:1000) of Bax (Bax Rabbit mAb, ABclonal Technology, Wuhan, China), GAPDH (GAPDH Rabbit mAb, ABclonal Technology, Wuhan, China), incubated at room temperature for 20 min, and after TBST membrane washing, 3 mL of secondary antibody (CST Company, Fig. USA) TBST dilute solution (1:2000), incubate at room temperature for 20 min, wash the TBST membrane and soak the PVDF membrane into TBST solution to prepare E CL chemiluminescence solution (Millipore, USA), drop it on the target protein strip of PVDF membrane, incubate at room temperature for 2 min in the dark, Images of the bands were collected using a chemiluminescence system (Tanon 4600, Shanghai Tianneng Technology Co. Ltd., Shanghai, China) and the gray values of the bands were quantified using Image J software.

### 2.7 Data Analysis

Quantitative data, including hormone levels and organ weights, were analyzed using GraphPad Prism 10. Multiple sample means were compared among groups, which met normal distribution and homogeneity of variance. One‐way ANOVA test was used for group comparisons when assumptions of normality and homogeneity of variance were met, with the Dunnett method used for pairwise comparisons. In cases with variance heterogeneity, the Welch test was employed, followed by Dunnett's T3 method for multiple comparisons. For non‐normally distributed data and count data, such as the number of follicles and follicular atresia rate, the Kruskal–Wallis H test (rank sum test) was conducted, with the Dunnett method for subsequent pairwise comparison. All data were collected and expressed as mean value $\bar{x}$ ± s, and *p <* 0.05 was considered statistically significant. Detailed full statistical outputs including all ANOVA and Kruskal–Wallis results are provided in Supplementary File S1.

## 3 Results

### 3.1 Changes in General Condition, Body Weight, and Estrous Cycle of Rats

Figure 2 a shows the design flow of the entire experiment. During the whole experiment, the rats in Group C were in good mental state, agile, smooth fur, and sensitive response; the rats in Group M increased with the modeling time, gradually developed decreased activity, apathy, disorganized yellowing of fur, and body weight was significantly decreased on the day of sacrifice compared with Group C (*p <* 0.01) (Figure 2b,c); Compared with rats in Group M, rats in Group P were more agile after a period of administration, with slightly shiny fur, but clearly showed irritability and a tendency to decrease body weight, but there was no significant difference (*p* > 0.05) (Figure 2b,c); After a period of administration, the rats in Groups ORL and ORH showed good mental status, smooth fur, and relatively increased activity. When compared with the M group, there was no significant difference in body weight (*p* > 0.05), but it was still significantly lower than that in the C group (*p <* 0.01). (Figure 2b,c). The estrous cycle of rats in Group C was stable, and the estrous cycle was stable at about 4 days per cycle. Rats in Group M showed estrous cycle disturbance, and nearly half (4 rats) of rats had disappearance of estrous cycle. Vaginal smear showed that they were in metestrus and diestrus for a long time (postestrus and dioestrus in Figure 2e), and some rats showed prolonged or shortened estrous cycle, consistent with the clinical manifestations of POF. Rats in Group P did not show loss of estrous cycle, and the number of secretions and cells in the vaginal smear on the day of estrus was high, and half (5 rats) of the rats had prolonged estrous cycle to 5 days, which was considered to be possibly related to the intervention of natural estrous cycle by the administration method of 5‐day cycle of estrogen and P in the simulated estrous cycle. Estrous cycles disappeared in 2 and 1 rats in ORL and ORH groups, respectively, and tended to shorten with increasing doses of OR administered (Figure 2d).

### 3.2 Changes in Serum Estrogen Levels

All blood samples for hormone level measurements were collected during the proestrus phase in each group of rats. Compared with Group C, serum E_2_ and P levels in Group M were significantly reduced (*p <* 0.01), LH activity was significantly increased (*p <* 0.05), and FSH activity was significantly elevated (*p <* 0.01), which is consistent with the clinical characteristics of POF.

**FIGURE 3:**
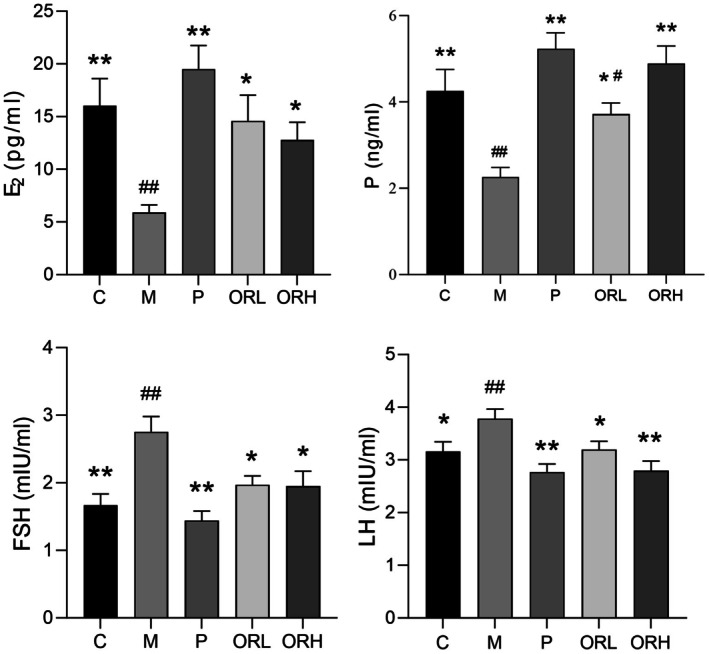
Effects of OR on sex hormones in serum of rats. (a) E2 levels. (b) P levels. (c) FSH levels. (d) LH levels. C, control group; M, model group; ORH, high‐dose OR group; ORL, low‐dose OR group; P, positive drug group. Data are represented as the mean ± SD from 10 animals in each group. *p < 0.05 and **p < 0.01 versus M group, ^#^ *p* < 0.05 and ^##^ *p* < 0.01 versus P group.

Compared with Group M, Group P showed a significant increase in serum E_2_ and P levels (*p <* 0.01) and a significant reduction in FSH and LH activities (*p <* 0.01). Group ORL exhibited a significant increase in serum E_2_ and P levels (*p <* 0.05) and a significant decrease in FSH and LH activities (*p <* 0.05). Group ORH also showed a significant increase in serum E_2_ levels (*p <* 0.05), a marked elevation in P levels (*p <* 0.01), a significant reduction in LH activity (*p* < 0.01), and a significant decrease in FSH activity (*p <* 0.05). These results (Figure 3) suggest that OR, particularly at higher doses, contributes to the restoration of hormone balance in POF rats, which may provide potential benefits.

### 3.3 Changes in Uterine and Ovarian Weights and Organ Index

**FIGURE 4:**
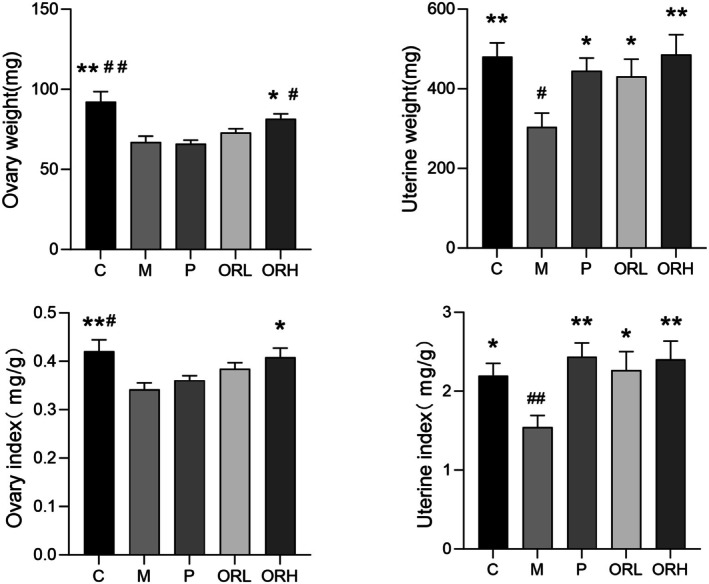
Effect of OR on ovarian weight, ovarian index, uterine weight, and uterine index (a) Ovarian weight of rats in each group. (b) Uterine weight of rats in each group. (c) Ovarian index of rats in each group. (d) Uterine index of rats in each group. C, control group; M, model group; ORH, high‐dose OR group; ORL, low‐dose OR group; P, positive drug group. Data are represented as the mean ± SD from 10 animals in each group. *p < 0.05 and **p < 0.01 versus M group, ^#^ *p* < 0.05 and ^##^ *p* < 0.01 versus P group.

All uterine and ovarian samples were collected during the proestrus phase. Compared with Group C, ovarian weight, ovarian index, and uterine weight in Group M were significantly reduced (*p <* 0.01), and the uterine index was also markedly decreased (*p <* 0.05), which is consistent with the clinical characteristics of POF (Figure 4). When compared with the M group, the uterine weight of rats in the P group was significantly increased (*p <* 0.05), and the uterine index was significantly increased (*p <* 0.01), but the ovarian index was still significantly lower than that of the control group (*p <* 0.01) (Figure 4). In the ORL group, the uterine weight and uterine index of rats were significantly increased (*p <* 0.05), but the ovarian weight was still significantly lower than that of the control group (*p <* 0.01) (Figure 4). In the ORH group, the ovarian weight and index of rats were significantly increased (*p <* 0.05), and the uterine weight and index were significantly increased (*p <* 0.01) (Figure 4).

### 3.4 Effects on Uterine Morphology and Endometrial Thickness in Rats

**FIGURE 5:**
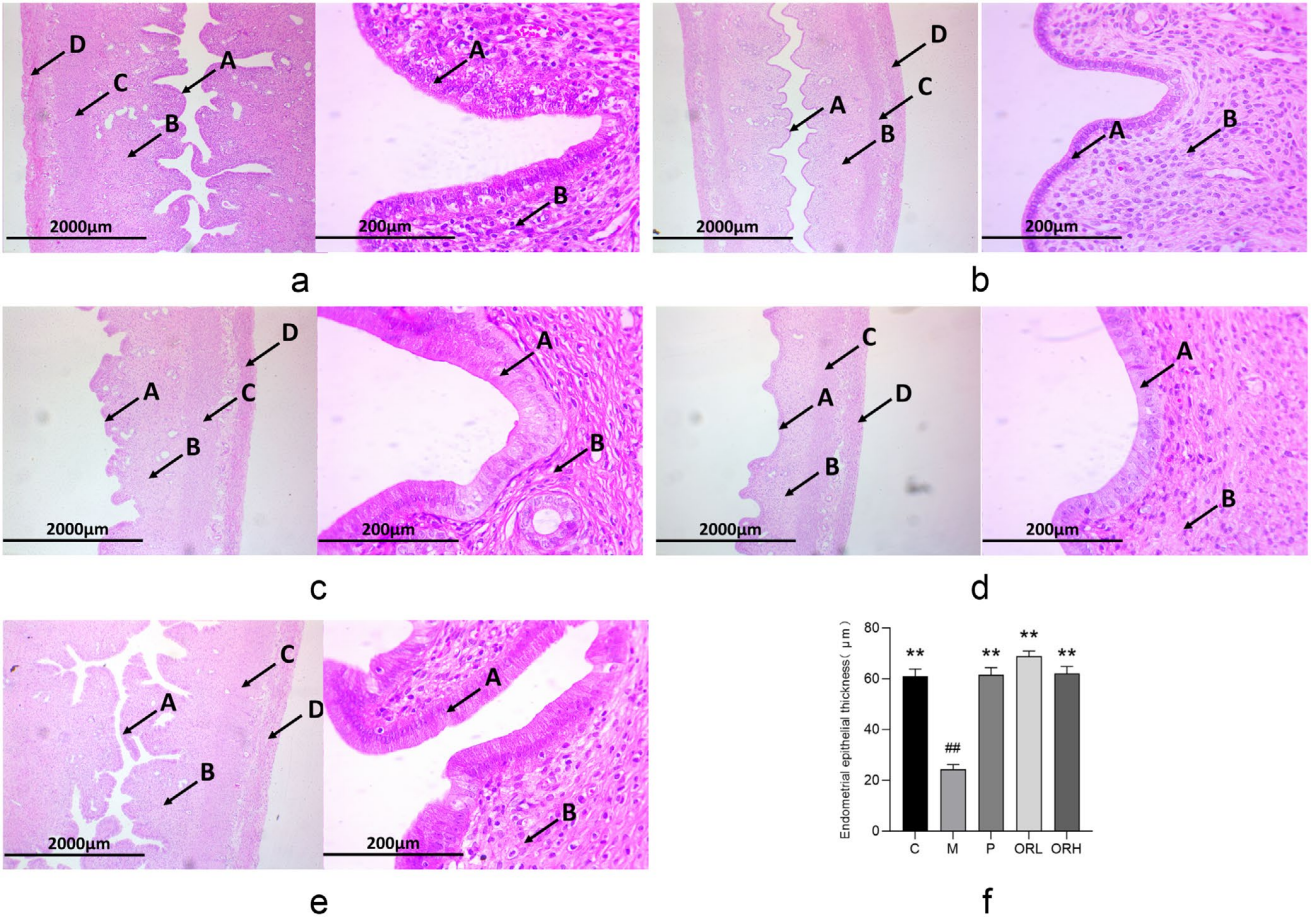
H&E staining of uterus and endometrial epithelial cell layer thickness. (a–e) Uterine tissue under 40× magnification and endometrial epithelial cell layer under 400×magnification in Groups C, M, P, ORL, and ORH are shown respectively, where arrow A points to the endometrial epithelial cell layer, arrow B points to the endometrial layer, arrow C points to the muscular layer, and arrow D points to the serosa layer. (f) endometrial epithelial cell layer thickness of rats in each group. C, control group; M, model group; ORH, high‐dose OR group; ORL, low‐dose OR group; P, positive drug group. Data are represented as the mean ± SD from 10 animals in each group. *p < 0.05 and **p < 0.01 versus M group, ^#^ *p* < 0.05 and ^##^ *p* < 0.01 versus P group.

All uterine samples were collected during the proestrus phase. In Group C, the endometrial, myometrial, and serosal layers were clearly distinguishable. The endometrial epithelium consisted of a single layer of columnar epithelial cells, with visible glands and blood vessels within the endometrial layer. The myometrium was composed of smooth muscle cells, and the outer serosal layer was smooth (Figure 5a). Compared with Group C, Group M exhibited uterine atrophy, with the endometrial epithelial cells transforming into a single layer of cuboidal epithelium, leading to a significant reduction in epithelial thickness (*p <* 0.01, Figure 5f). Additionally, endometrial stromal atrophy was observed, with a reduction in gland numbers, loosely arranged cells, and myometrial atrophy, although the serosal layer remained unchanged (Figure 5b).

Compared with Group M, Group P showed a restoration of the endometrial epithelium to a single layer of columnar cells, with a significant increase in epithelial thickness (*p <* 0.01, Figure 5f). The endometrial stroma exhibited no signs of atrophy, with an increase in gland numbers and a more compact cellular arrangement, and slight improvement in myometrial atrophy (Figure 5c). In Group ORL, the endometrial epithelium also recovered to a single layer of columnar cells, with a significant increase in epithelial thickness (*p <* 0.01, Figure 5f), but endometrial stromal atrophy showed only slight improvement, and gland numbers remained unchanged (Figure 5d). In Group ORH, the endometrial epithelium was restored to a single layer of columnar cells, with a significant increase in epithelial thickness (*p <* 0.01, Figure 5f), improved endometrial stromal atrophy, a slight increase in gland numbers, and slight improvement in myometrial atrophy (Figure 5e).

### 3.5 Effects on Follicle Counts and Atresia Rates at Different Developmental Stages

**FIGURE 6:**
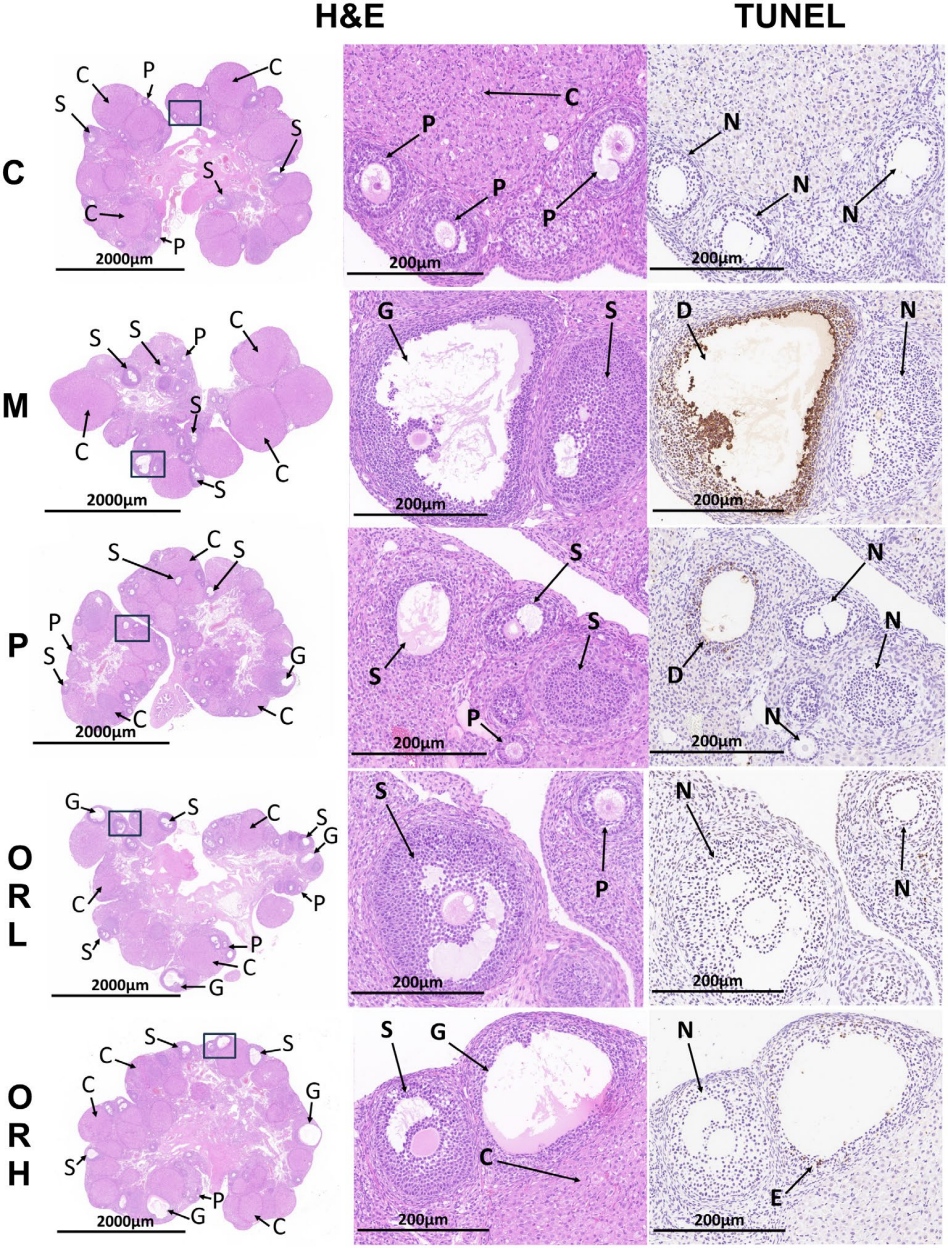
H&E and TUNEL staining of ovaries. Continuous sections of the ovaries were stained with H&E and TUNEL respectively, which can observe follicles at different developmental stages and apoptosis of follicle cells. The high‐power image of the ovary is an enlargement of the insert in the low‐power image, with scale bars of 200 and 2000 μm, respectively. In H&E staining figure, the C arrows indicate corpora lutea, the P arrows indicate primary follicles, the S arrows indicate secondary follicles and the G arrows indicate Graafian follicles. In TUNEL staining figure, the E arrow points to the outer granule cells of follicles with small amount of apoptosis, the D arrow points to atresia follicles with apoptosis of large numbers of granule cells, the N arrows point to normally developing follicles.

Histological analysis of H&E‐stained ovarian sections revealed multiple stages of follicular development, including primordial follicles, primary follicles, secondary follicles with distinct antral cavities, Graafian follicles with a well‐defined cumulus‐oocyte complex, and newly formed corpora lutea. The cortical region was supported by fibrous connective tissue, and the medullary region contained abundant blood vessels. TUNEL‐stained ovarian sections showed clearly identifiable atretic follicles or follicles undergoing atresia (Figure 6).

When compared with Group C, Group M showed statistically significant results as follows: the number of primary follicles increased (*p <* 0.05), while the counts of secondary follicles and Graafian follicles decreased significantly (*p <* 0.05). Notably, the number of corpus luteum in Group M was markedly reduced (*p <* 0.01). Additionally, Group M exhibited a significant rise in the atresia rates of primary follicles and Graafian follicles (*p <* 0.01), along with a notable increase in the atresia rate of secondary follicles (*p <* 0.05).

When compared with Group M, the ovaries of rats in Group P showed a significant increase in the number of secondary follicles, Graafian follicles, and corpus lutea (all *p <* 0.01). Notably, the number of secondary follicles in Group P even exceeded that in the control group (*p <* 0.01). Additionally, Group P exhibited a significant decrease in the atresia rate of primary follicles (*p <* 0.05), though it remained significantly higher than the control group (*p <* 0.01).

In Group ORL, the counts of secondary follicles, Graafian follicles, and corpus lutea were all significantly increased (*p <* 0.05). The atresia rate of primary follicles was notably reduced (*p <* 0.01), but still remained significantly higher than the C group (*p <* 0.01). Moreover, the atresia rates of secondary and Graafian follicles were significantly decreased (*p <* 0.05). For Group ORH, the numbers of secondary follicles and Graafian follicles were significantly elevated (*p <* 0.05), while the count of corpus lutea showed a marked increase (*p <* 0.01). The atresia rates of primary follicles were significantly reduced (*p <* 0.01), but the latter still remained higher than the C group (*p <* 0.01). Additionally, the atresia rates of secondary and Graafian follicles were significantly decreased (*p <* 0.05). (Table 1).

**TABLE 1:** Effects of OR on follicles at various stages of development in the ovary of rats.

Group	Primary follicle (EA)	Secondary follicle (EA)	Graafian follicle (EA)	Corpora lutea (EA)	Atresia rate of primary follicle (%)	Atresia rate of secondary follicle (%)	Atresia rate of Graafian follicle (%)
C	13.20 ± 3.49*^#^	13.50 ± 2.18*^##^	2.20 ± 0.42*	10.50 ± 2.17**	10.61 ± 1.83**^##^	47.40 ± 15.14*^#^	18.18 ± 5.06**^##^
M	17.50 ± 4.05	7.60 ± 1.29^##^	1.20 ± 0.48^##^	6.70 ± 0.82^##^	53.14 ± 9.35	84.21 ± 29.85	50.00 ± 17.13
P	18.80 ± 6.33	20.00 ± 7.35**	2.50 ± 0.59**	11.70 ± 1.86**	34.57 ± 4.13*	75.00 ± 24.03	44.00 ± 15.33
ORL	15.00 ± 3.77	12.40 ± 3.78*^##^	2.10 ± 0.39*	9.70 ± 2.16*	25.33 ± 3.77**^#^	51.61 ± 17.33*^#^	28.57 ± 10.44*
ORH	14.50 ± 3.81	11.50 ± 2.23*^##^	2.30 ± 0.45*	12.00 ± 2.10**	23.45 ± 3.40**^#^	50.43 ± 16.90*^#^	30.43 ± 11.39*

*Note:* Data are represented as the mean ± SD from 10 animals in each group. *p < 0.05 and **p < 0.01 versus M group, ^#^ *p* < 0.05 and ^##^ *p* < 0.01 versus P group.

Abbreviations: C, control group; M, model group; ORH, high‐dose OR group; ORL, low‐dose OR group; P, positive drug group.

### 3.6 Effects on Bcl2 and Bax Protein Expression in Rat Ovaries

**FIGURE 7:**
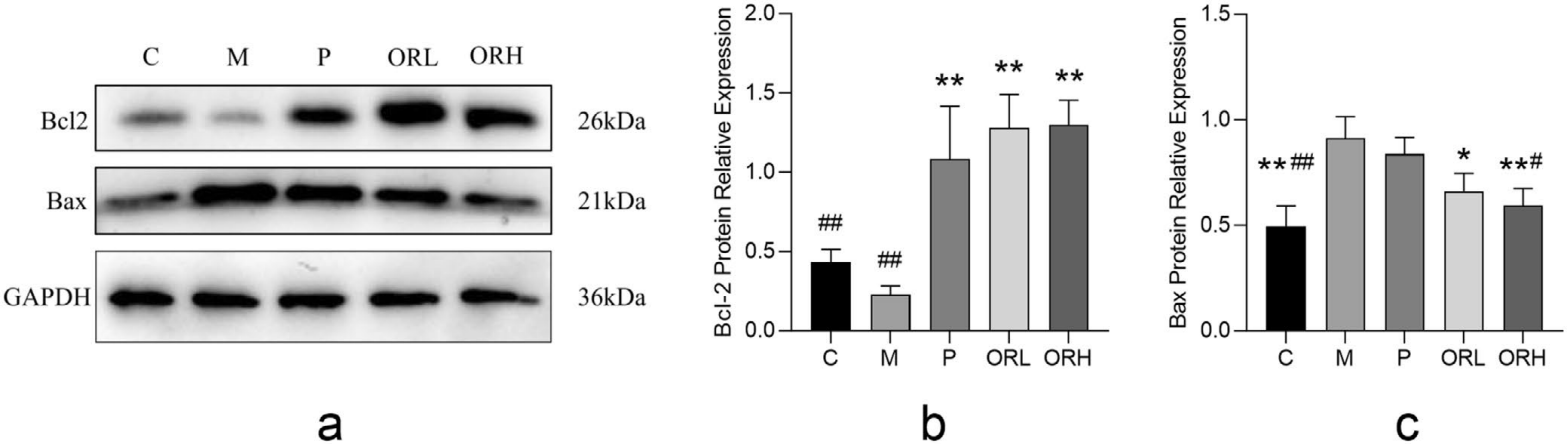
Effects of OR on protein expression of Bcl2 and Bax proteins in the ovary of rats. (a) Indicated representative immunoblots of Bcl2 and Bax proteins in the ovaries of each group. (b) Shows the expression of Bcl2 protein relative to GAPDH protein. (c) Shows the expression of Bax protein relative to GAPDH protein. C, control group; M, model group; ORH, high‐dose OR group; ORL, low‐dose OR group; P, positive drug group. Data are represented as the mean ± SD from 3 animals in each group. *p < 0.05 and **p < 0.01 versus M group, ^#^ *p* < 0.05 and ^##^ *p* < 0.01 versus P group. Full uncropped blots for this panel are presented in Supplementary File S2, Figure S1–S5.

Compared with Group C, Bax protein expression in the ovaries of Group M was significantly increased (*p <* 0.01). Compared with Group M, Bcl‐2 protein expression was significantly upregulated in Group P (*p <* 0.01). In Group ORL, Bcl‐2 protein expression was markedly increased (*p <* 0.01), while Bax protein expression was significantly decreased (*p <* 0.05). In Group ORH, Bcl‐2 protein expression was significantly upregulated (*p <* 0.01), whereas Bax protein expression was markedly downregulated (*p* < 0.01) (Figure 7). All full uncropped Western blot membranes corresponding to Figure 7 are provided in Supplementary File S2 (Figures S1–S5).

### 3.7 Effect on PI3K, Akt, p‐Akt, and PTEN Protein Expression in Rat Ovaries

**FIGURE 8:**
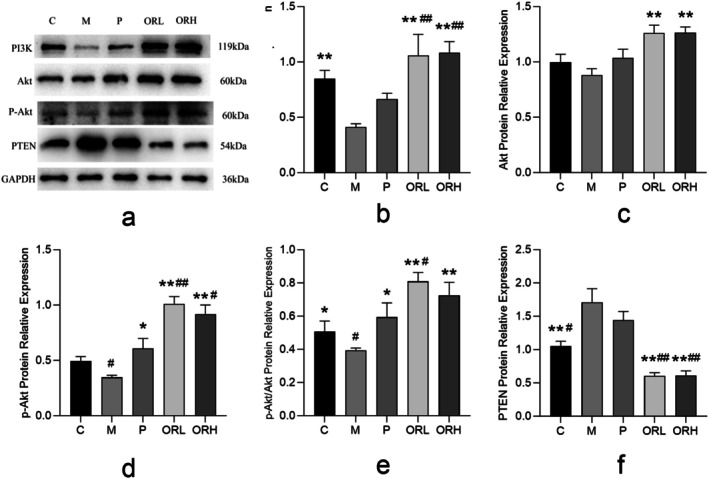
Effects of OR on protein expression of PI3K, Akt, p‐Akt, and PTEN proteins in the ovary of rats. (a) Indicated representative immunoblots of PI3K, Akt, p‐Akt, and PTEN proteins in the ovaries of each group. (b) Shows the expression of PI3K protein relative to GAPDH protein. (c) Shows the expression of Akt protein relative to GAPDH protein. (d) Shows the expression of p‐Akt protein relative to GAPDH protein. (e) Shows the expression of p‐Akt protein relative to Akt protein. (f) Shows the expression of PTEN protein relative to GAPDH protein. C, control group; M, model group; ORH, high‐dose OR group; ORL, low‐dose OR group; P, positive drug group. Data are represented as the mean ± SD from 3 animals in each group. *p < 0.05 and **p < 0.01 versus M group, ^#^ *p* < 0.05 and ^##^ *p* < 0.01 versus P group. Full uncropped blots for this panel are presented in Supplementary File S2 (Figure S6–S12).

Compared with Group C, PI3K protein expression in the ovaries of Group M was significantly reduced (*p <* 0.01), Akt phosphorylation levels were notably decreased (*p <* 0.05), and PTEN protein expression was significantly increased (*p <* 0.01). Compared with Group M, p‐Akt protein expression in Group P was significantly increased (*p <* 0.05), along with a notable elevation in Akt phosphorylation levels (*p <* 0.05). In Group ORL, PI3K, Akt, and p‐Akt protein expression were all significantly upregulated (*p <* 0.01), accompanied by a marked increase in Akt phosphorylation levels (*p <* 0.01) and a significant reduction in PTEN protein expression (*p <* 0.01). Similarly, in Group ORH, PI3K, Akt, and p‐Akt protein expression were significantly increased (*p <* 0.01), with a notable elevation in Akt phosphorylation levels (*p <* 0.01) and a significant decrease in PTEN protein expression (*p <* 0.01) (Figure 8). All full uncropped Western blot membranes corresponding to Figure 8 are provided in Supplementary File S2 (Figures S6–S12).

## 4 Discussion

In this study, the effect of OR on POF in rats was evaluated. A rat model of POF was established by intraperitoneal injection of CTX. Female rats showed a series of symptoms consistent with the clinical manifestations of POF, such as estrous cycle disorder, decreased estrogen level, increased hormone level, ovarian and uterine atrophy, decreased number of follicles in the ovary, and increased atresia rate. OR treatment significantly alleviated estrous cycle disturbance, elevated estrogen levels, reduced gonadotropins, restored uterine atrophy, thickened the endometrial epithelium, increased ovarian weight, and enhanced the number of secondary and Graafian follicles while reducing their atresia rates. These changes may be mediated by OR up‐regulating the PI3K‐Akt signaling pathways and Bcl‐2/Bax apoptotic axis in the ovary and exerting an “estrogen‐like effect” by improving follicular status. ERT combined with ovulation induction improved most symptoms, but did not improve secondary and Graafian follicles atresia. Considering the increase in the number of secondary and Graafian follicles in the ovary, ovulation induction with clomiphene citrate may cause ovarian hyperstimulation, which may exacerbate POF in the long‐term.

Notably, the present study did not observe a strict linear dose–response relationship between low‐ and high‐dose OR for several key ovarian and uterine endpoints. Based on our previous in vitro experiments, our research group speculates that the material basis responsible for the estrogen‐like effect of OR may involve certain functional oligopeptides. These bioactive peptides are thought to be generated gradually through digestion by gastrointestinal enzymes and biotransformation by the gut microbiota, rather than existing as fully active forms in the raw material. Furthermore, OR forms a stable gelatinous matrix after water reconstitution, which slows its disintegration, swelling, and proteolytic release in the gastrointestinal tract. At high doses, this viscous gel may create a diffusion barrier that limits complete digestion and absorption, leading to saturation of bioactivation rather than further enhancement of biological effects. By contrast, the low dose appears better matched to the digestive capacity and bioactivation efficiency of rats, thereby achieving near‐maximal physiological effects. Thus, the lack of a pronounced dose dependency does not contradict the protective efficacy of OR, but instead reflects its unique gastrointestinal bioactivation pattern and dose‐efficient utilization profile.

In this study, CTX was injected intraperitoneally to establish a rat model of POF. CTX is an antineoplastic agent of the alkylating agent class that has toxic effects on the reproductive system, particularly the ovary. CTX activates primordial follicles in large numbers, but these follicles are largely atretic at the primary follicular stage, resulting in a decrease in primordial follicle reserve in the ovary (Kalich‐Philosoph et al. 2013). CTX also damages granulosa cells, causing secondary and Graafian follicular atresia; notably, long‐term use can cause permanent damage to the ovary, greatly increasing the risk of POF (Mouridsen et al. 1976; Nguyen et al. 2019). The use of CTX can cause menstrual cycle disorders, decreased estrogen levels, and increased hormone levels, which are similar to the symptoms of POF. Therefore, CTX is applied to prepare POF models, but the dose and cycle of CTX have not been standardized. In the preliminary experimental stage, the appearance, body weight, and organ weight of female rats were measured after a single injection of CTX 100–200 mg/kg. After a single injection of CTX 100 mg/kg, the body weight of rats first rapidly decreased and then quickly recovered; no significant difference in ovarian weight and uterine weight was observed compared with the control group after 14 days. After a single injection of CTX 200 mg/kg, the body weight of some rats decreased to less than 150 g, and animal deaths occurred. In addition to reproductive toxicity, CTX also suppresses the digestive system and the bone marrow, leading to reduced immunity (De Flora and Ferguson 2005). Thus, an excessive dose of CTX should be avoided. Hence, the basal dose of 100 mg/kg was injected, followed by 10 mg/kg supplemented daily to prepare the POF model (Nie et al. 2021). At 14 days of modeling, the measurement parameters of the rats were in accordance with the clinical symptoms of POF, while the appearance and body weight remained in a relatively safe range. No animal death occurred at this dose, so this method was selected to prepare the POF model.

In women, antral follicles in the ovaries undergo periodic growth and maturation in response to pituitary gonadotropins throughout the menstrual cycle, leading to fluctuations in estrogen levels. Follicles in the ovaries originate as primordial follicles and undergo a long growth process into primary and secondary follicles, lasting approximately 200 days in humans and 58 days in rats (Gougeon 1996) to form antral follicles. These are characterized by an abundance of FSH receptors in the internal granulosa cells (Celik et al. 2008), which are regulated by pituitary gonadotropins and grow and mature in response to the menstrual cycle, which takes about 14 days in humans and 2–3 days in rats (Gougeon 1996). The activation and growth of follicles from the resting state are regulated by the PI3K signaling pathway (Zhang and Liu 2015), with PTEN serving as a key negative regulator (Reddy et al. 2008). PI3K can phosphorylate PIP2 to PIP3, and the PH region of Akt can bind to PIP3, inducing Akt translocation from the cytosol. The transferred Akt is completely phosphorylated on the cell membrane, and phosphorylated Akt causes a cascade of signal transduction pathways (Grosbois and Demeestere 2018). PTEN can dephosphorylate PIP3 to PIP2. This hinders the PI3K‐Akt signaling pathway, resulting in decreased PIP3 content (Mirza‐Aghazadeh‐Attari et al. 2020). In our preliminary study (He et al. 2023), transcriptomics technology was used to investigate the changes in differentially expressed genes in the ovaries of POF rats after intervention with OR. The results demonstrated that 289 differentially expressed genes (DEGs) were identified in the ovaries following OR administration, which were enriched in 47 signaling pathways. Key pathways identified included the PI3K/Akt signaling pathway, osteoclast differentiation, viral myocarditis, B‐cell receptor signaling pathway, among others. These findings were confirmed in the present study, indicating that the PI3K‐Akt signaling pathway is upregulated in the ovaries of POF rats after administration of OR. Furthermore, activation of this signaling pathway led to an increase in the number of antral follicles and corpora lutea in the ovaries, resulting in higher levels of secreted estrogen and progesterone.

Menopause occurs when the reserve of primordial follicles decreases or is completely depleted. In POF patients, this process occurs prematurely, as the ovaries frequently exhibit atresia of antral follicles, leading to anovulation and infertility. In multiparous rats, this manifests as increased antral follicle atresia rate and reduced litter size. Follicular atresia is a spontaneous, genetically regulated process of programmed cell death, although the exact triggers remain unclear (Worku et al. 2017). The “outer granulosa cell damage hypothesis” is currently one of the leading theories explaining follicular atresia (Hułas‐Stasiak and Gawron 2011). TUNEL staining showed extensive follicular atresia in POF rats, with obvious DNA damage and granulosa cell apoptosis, especially in the outer layer, consistent with previous studies (Nie et al. 2020; Huang et al. 2020). Bax and Bcl‐2 proteins belong to the Bcl‐2 family and regulate mitochondrial membrane permeability to determine whether the apoptotic process is activated. Both Bax and Bcl‐2 proteins are regulated by the PI3K/Akt signaling pathway. AKT can directly phosphorylate the proapoptotic protein Bad at the Ser136 site, causing it to dissociate from the Bcl‐2/Bcl‐xL complex and release antiapoptotic Bcl‐2 with activity. Bcl‐2 is an antiapoptotic protein that functions primarily by inhibiting proapoptotic factors like Bax, thereby protecting cells from apoptosis (Renault et al. 2017; Hafezi and Rahmani 2021). Bax is a proapoptotic protein that increases mitochondrial membrane permeability, leading to the release of cytochrome c and other apoptotic factors into the cytoplasm, thereby triggering apoptosis (Spitz and Gavathiotis 2022). In this study, administration of OR induced the up‐regulation of Bcl‐2 protein expression, while Bax protein expression was downregulated in the ovaries of POF rats. Consequently, the follicular atresia rate, which is regulated by these proteins, was significantly reduced.

In this study, the treatment administered to rats in the P group simulated the clinical management of POF patients seeking fertility, namely ERT combined with ovulation induction. The results demonstrated that OR exerted therapeutic effects similar to ERT plus ovulation induction in restoring hormonal balance, improving uterine and ovarian morphology, and increasing the number of secondary and mature follicles. Nevertheless, important differences may exist between the two treatments in their mechanisms of action and potential risk profiles, providing distinct considerations for clinical translation. Clinically, ERT directly corrects estrogen deficiency through exogenous supplementation, and clomiphene is subsequently used to stimulate the development of numerous antral follicles to improve the chance of successful ovulation. In contrast, OR may improve intrinsic ovarian function and follicle reserve by upregulating the endogenous PI3K‐Akt survival signaling pathway and inhibiting granulosa cell apoptosis through regulating the expression balance of Bcl‐2 and Bax, thereby reducing the follicular atresia rate. Although the number of secondary follicles in the OR group was significantly lower than that in the P group, OR significantly decreased the follicular atresia rate and achieved a number of mature follicles comparable to that of the P group (Table 1). These findings suggest that OR may possess unique advantages in directly protecting follicles and delaying the depletion of ovarian reserve.

Long‐term ERT is known to be associated with an increased risk of breast cancer, endometrial cancer, and other adverse outcomes (Rossouw et al. 2013). As a historically recognized medicinal and edible homologous substance, OR has been shown to possess a high safety profile in previous toxicity studies, with no obvious pathological changes observed in major target organs following a 28‐day feeding test (Zhang et al. 2017). Regarding cancer risk assessment, one study detected TβRI and TβRII, two endometrial cancer‐related proteins, in the uterus and found no abnormal expression (Kang et al. 2015). In our preliminary research, OR administration was found to upregulate follicular FSHR protein expression (Zhao et al. 2024). Prolonged OR treatment may induce mild, transient ovarian hyperstimulation similar to clomiphene. Therefore, excessive long‐term use should be applied cautiously. In summary, the follicle‐protective and endogenous ovarian function‐improving properties of OR indicate its potential as an adjuvant strategy for early‐stage POF patients who desire pregnancy or wish to delay ovarian function decline. It may represent a promising alternative for patients unwilling or unsuitable to receive long‐term ERT.

This study elucidated the beneficial effects and potential mechanisms of OR in POF rats. However, the limitations of the present study should be acknowledged. First, the present study only detected changes in the expression levels of proteins related to the PI3K/Akt signaling pathway and the Bcl‐2/Bax apoptotic axis but did not use specific pathway inhibitors or agonists for reverse validation. Therefore, a definitive causal relationship between OR‐mediated regulation of these pathways and the improvement of ovarian function cannot be fully confirmed in the current study. In addition, the upstream molecular targets through which OR acts to regulate these signaling cascades remain to be further identified and verified. Previous studies have reported that the addition of OR‐containing serum to culture media can protect granulosa cells from ROS‐induced damage and inhibit apoptosis, potentially involving the activation of the mitogen‐activated protein kinase (MAPK) signaling pathway (Ling et al. 2017). Future research should focus on analyzing the protective effects of OR on granulosa cells to further elucidate its role in preventing follicular atresia. Second, while OR demonstrated benefits in POF rats, its effects in human POF patients remain uncertain. The menstrual cycle in humans is 5–7 times longer than the estrous cycle in rats, and typically, only one antral follicle in each menstrual cycle acts as the “dominant follicle” and matures and ovulates, while the remaining follicles undergo spontaneous atresia. In contrast, rats develop multiple dominant follicles per estrous cycle, with young female rats capable of ovulating more than 15 oocytes per cycle; consequently, rats have a lower follicular atresia rate compared to humans. Therefore, rats may be more sensitive to both the ovarian toxicity of CTX and the protective effects of OR. Hence, further clinical studies in humans are required to determine whether OR can provide therapeutic benefits for POF patients.

## 5 Conclusion

In summary, this study investigated the therapeutic effects of OR on CTX‐induced POF in rats. Administration of OR alleviated estrous cycle disorders, increased serum estrogen levels, decreased gonadotropin levels, restored uterine atrophy, increased endometrial epithelial thickness, improved ovarian weight, elevated the counts of secondary and Graafian follicles, and reduced follicular atresia rates. Mechanistically, these beneficial effects are correlated with OR‐induced modulation of the PI3K‐Akt survival pathway and the expression of apoptosis‐related proteins Bcl‐2 and Bax in ovarian tissue, which may contribute to improved follicular status and ovarian protection. Importantly, this study not only enriches the efficacy evidence supporting the potential use of OR for POF management, but also provides novel systematic evidence at the protein signaling level demonstrating that the protective effects of OR are associated with the activation of the PI3K‐Akt pathway and the inhibition of the mitochondrial apoptotic cascade regulated by Bcl‐2 and Bax. These findings provide new scientific support and a promising candidate for developing POF intervention strategies targeting ovarian signaling pathways. Further clinical investigations are warranted to determine whether similar benefits can be achieved in human POF patients.

## Author Contributions

**Hongyu Zhao:** conceptualization, data curation, funding acquisition, methodology, project administration, resources, writing – original draft. **Xingyao He:** data curation, investigation, software. **Lin Di:** supervision, resources. **Xianchun Cheng:** data curation, investigation, software, writing – review and editing. **Yu Wang:** conceptualization, data curation, formal analysis, methodology, writing – review and editing. **Zhen Yang:** writing – review and editing, resources, funding acquisition, project administration, supervision. **Xueyuan Sun:** software, data curation, investigation.

## Funding

This work was supported by the Natural Science Foundation of Jilin Province (YDZJ202201ZYTS264), Changchun Science and Technology Project (21ZY62), and Jilin Province Health Science and Technology ability Improvement Project (2023JC068). The funding did not have a role in the design of the study, the collection, analysis, and interpretation of data, or writing the manuscript.

## Ethics Statement

This study was reviewed and approved by the Laboratory Animal Ethics Committee of Jilin Academy of Chinese Medical Sciences (Approval No.: JLSZKYDWLL2021‐012) in accordance with the regulations of the Laboratory Animal Ethics Committee.

## Conflicts of Interest

The authors declare no conflicts of interest.

## Supporting information


**Table S1:** Results of one‐way ANOVA analyses.
**Table S2:** Results of Kruskal–Wallis analyses.

**Figure S1:** Original gel of Bax.
**Figure S2:** Bax‐merged images of the visible light image and chemiluminescence blot.
**Figure S3:** Original gel of Bcl2.
**Figure S4:** Bcl2‐merged images of the visible light image and chemiluminescence blot.
**Figure S5:** Original gel of GAPDH.
**Figure S6:** Original gel of PI3K.
**Figure S7:** PI3K‐merged images of the visible light image and chemiluminescence blot.
**Figure S8:** Original gel of AKT.
**Figure S9:** Original gel of p‐AKT.
**Figure S10:** Original gel of PTEN.
**Figure S11:** PTEN‐merged images of the visible light image and chemiluminescence blot.
**Figure S12:** Original gel of GAPDH.

## Data Availability

The data that support the findings of this study are available from the corresponding author upon reasonable request.
