# Supplementary material for: Oviductus Ranae as a Functional Food for Ovary Protection: Ameliorating Premature Ovarian Failure and Modulating PI3K/Akt and Apoptosis Pathways in Rats
Source: Food Sci Nutr. 2026 Jul 7;14(7):e72089. doi: 10.1002/fsn3.72089 (PMC13341967; doi:10.1002/fsn3.72089)
Supplement: Supplementary file 1 — Table S1: Results of one‐way ANOVA analyses. Table S2: Results of Kruskal–Wallis analyses. [file FSN3-14-e72089-s001.docx]

**Table 1 Results of One-way ANOVA Analyses**

| No. | Variable | df | F | P |
| --- | --- | --- | --- | --- |
| 1 | Sacrifice day body weight | 4,45 | 21.15 | 0.0000000007 |
| 2 | E2 | 4,45 | 7.565 | 0.00009 |
| 3 | P | 4,45 | 24.81 | 0.00000000007 |
| 4 | FSH | 4,45 | 20.48 | 0.000000001 |
| 5 | LH | 4,45 | 14.56 | 0.0000001 |
| 6 | Ovary weight | 4,45 | 8.165 | 0.00005 |
| 7 | Uterine weight | 4,45 | 3.433 | 0.015 |
| 8 | Ovary index | 4,45 | 3.927 | 0.008 |
| 9 | Uterine index | 4,45 | 3.562 | 0.013 |
| 10 | Endometrial epithelial thickness | 4,45 | 50.89 | ＜1×10^-15^ |
| 11 | Bcl-2 Protein Relative Expression | 4,10 | 19.85 | 0.0001 |
| 12 | Bax Protein Relative Expression | 4,10 | 11.28 | 0.001 |
| 13 | PI3K Protein Relative Expression | 4,10 | 20.99 | 0.00007 |
| 14 | Akt Protein Relative Expression | 4,10 | 6.223 | 0.009 |
| 15 | p-Akt Protein Relative Expression | 4,10 | 18.47 | 0.0001 |
| 16 | p-Akt/Akt Protein Relative Expression | 4,10 | 20.31 | 0.00009 |
| 17 | PTEN Protein Relative Expression | 4,10 | 51.21 | 0.000001 |

**Table 2 Results of Kruskal-Wallis Analyses**

| No. | Variable | df | H | P |
| --- | --- | --- | --- | --- |
| 1 | Primary Follicle | 4 | 9.4 | 0.048 |
| 2 | Secondary Follicle | 4 | 33.79 | 0.0000008 |
| 3 | Graafian Follicle | 4 | 28.37 | 0.00001 |
| 4 | Corpora lutea | 4 | 31.20 | 0.000003 |
| 5 | Atresia rate of Primary follicle | 4 | 44.27 | 0.000000006 |
| 6 | Atresia rate of Secondary follicle | 4 | 15.85 | 0.003 |
| 7 | Atresia rate of Graafian follicle | 4 | 24.98 | 0.00005 |
