# Supplementary material for: Oviductus Ranae as a Functional Food for Ovary Protection: Ameliorating Premature Ovarian Failure and Modulating PI3K/Akt and Apoptosis Pathways in Rats
Source: Food Sci Nutr. 2026 Jul 7;14(7):e72089. doi: 10.1002/fsn3.72089 (PMC13341967; doi:10.1002/fsn3.72089)
Supplement: Supplementary file 2 — Figure S1: Original gel of Bax. Figure S2: Bax‐merged images of the visible light image and chemiluminescence blot. Figure S3: Original gel of Bcl2. Figure S4: Bcl2‐merged images of the visible light image and chemiluminescence blot. Figure S5: Original gel of GAPDH. Figure S6: Original gel of PI3K. Figure S7: PI3K‐merged images of the visible light image and chemiluminescence blot. Figure S8: Original gel of AKT. Figure S9: Original gel of p‐AKT. Figure S10: Original gel of PTEN. Figure S11: PTEN‐merged images of the visible light image and chemiluminescence blot. Figure S12: Original gel of GAPDH. [file FSN3-14-e72089-s002.docx]

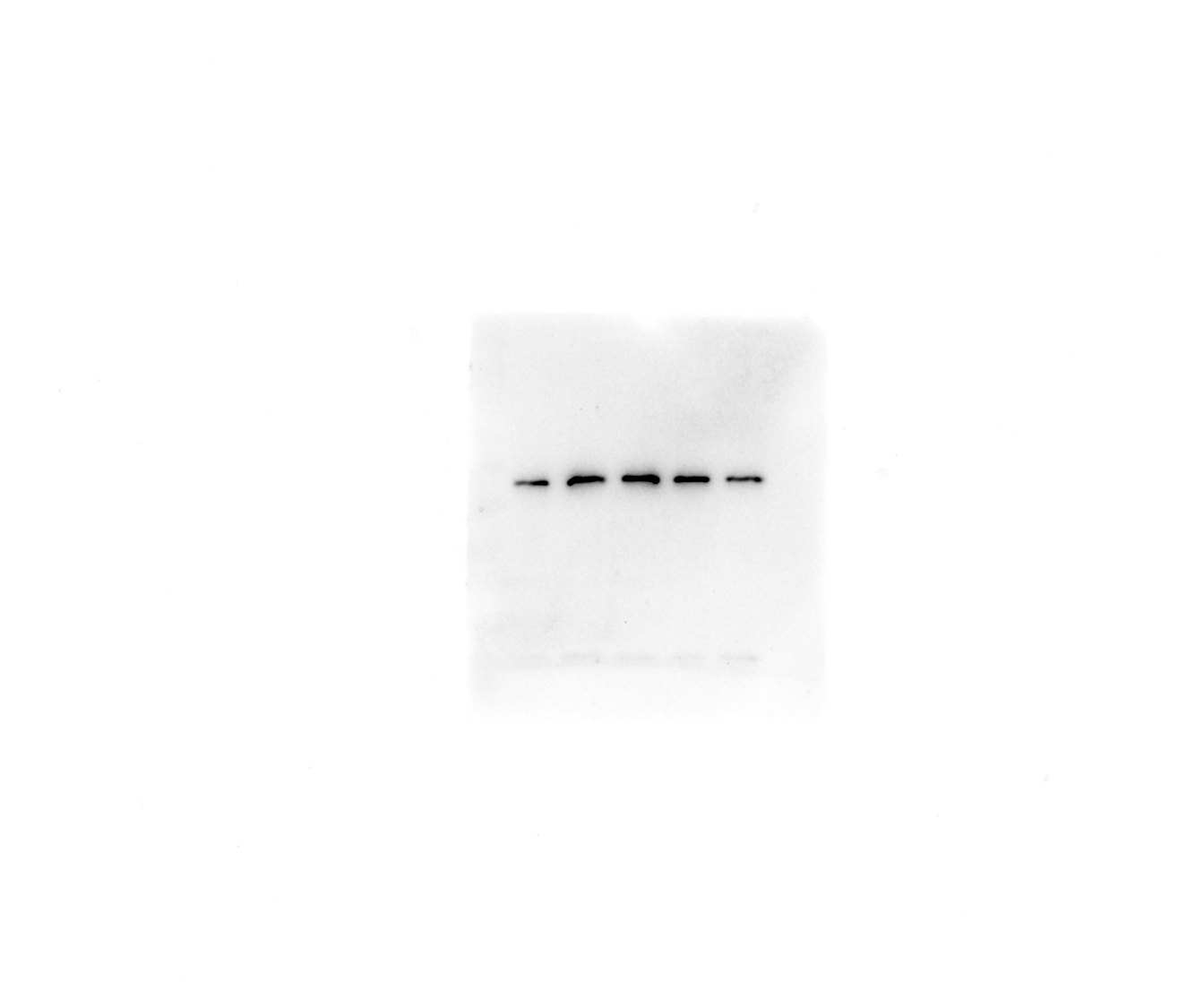


Fig S1 original gel of Bax


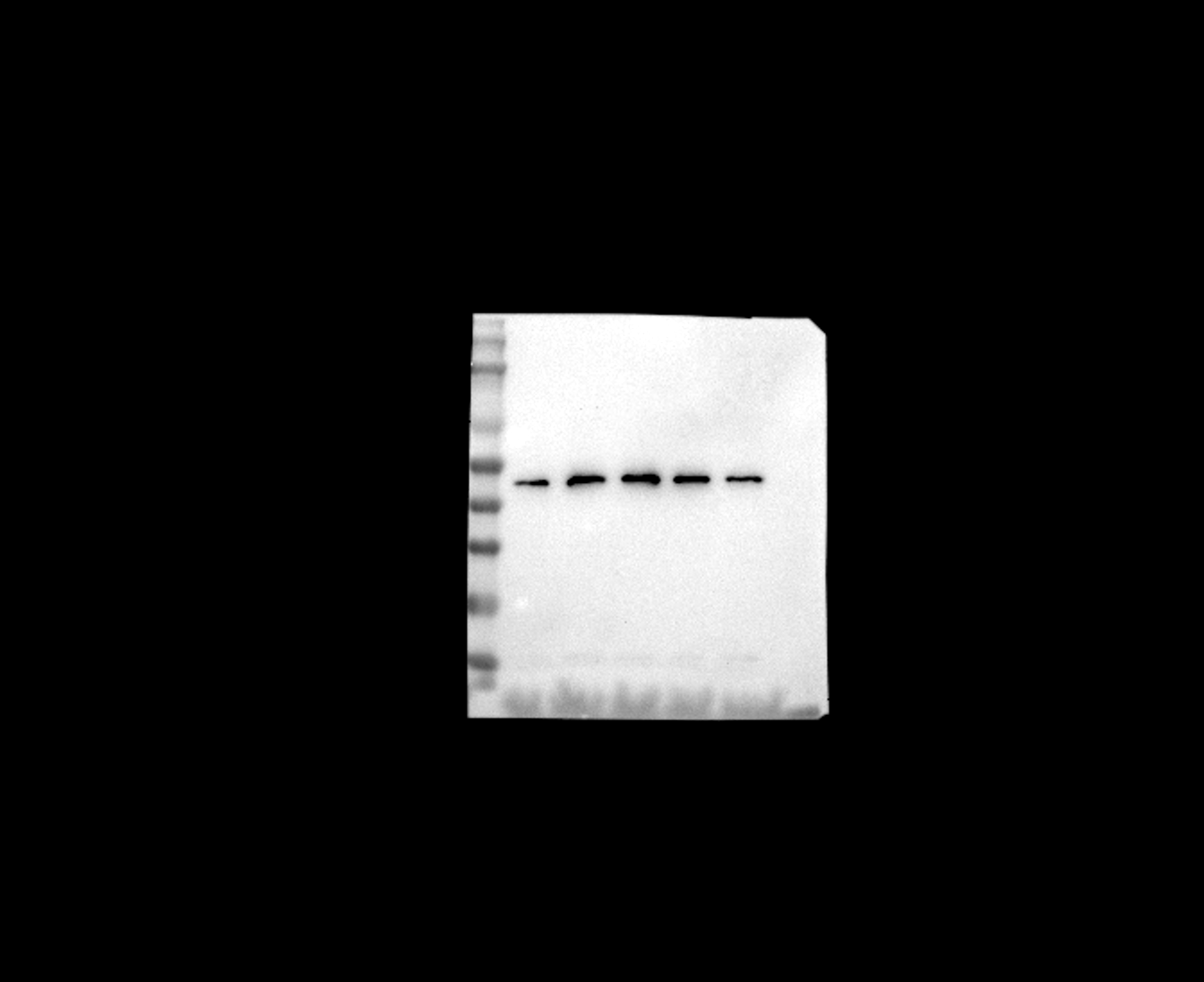


Fig S2 Bax-merged images of the visible light image and chemiluminescence blot


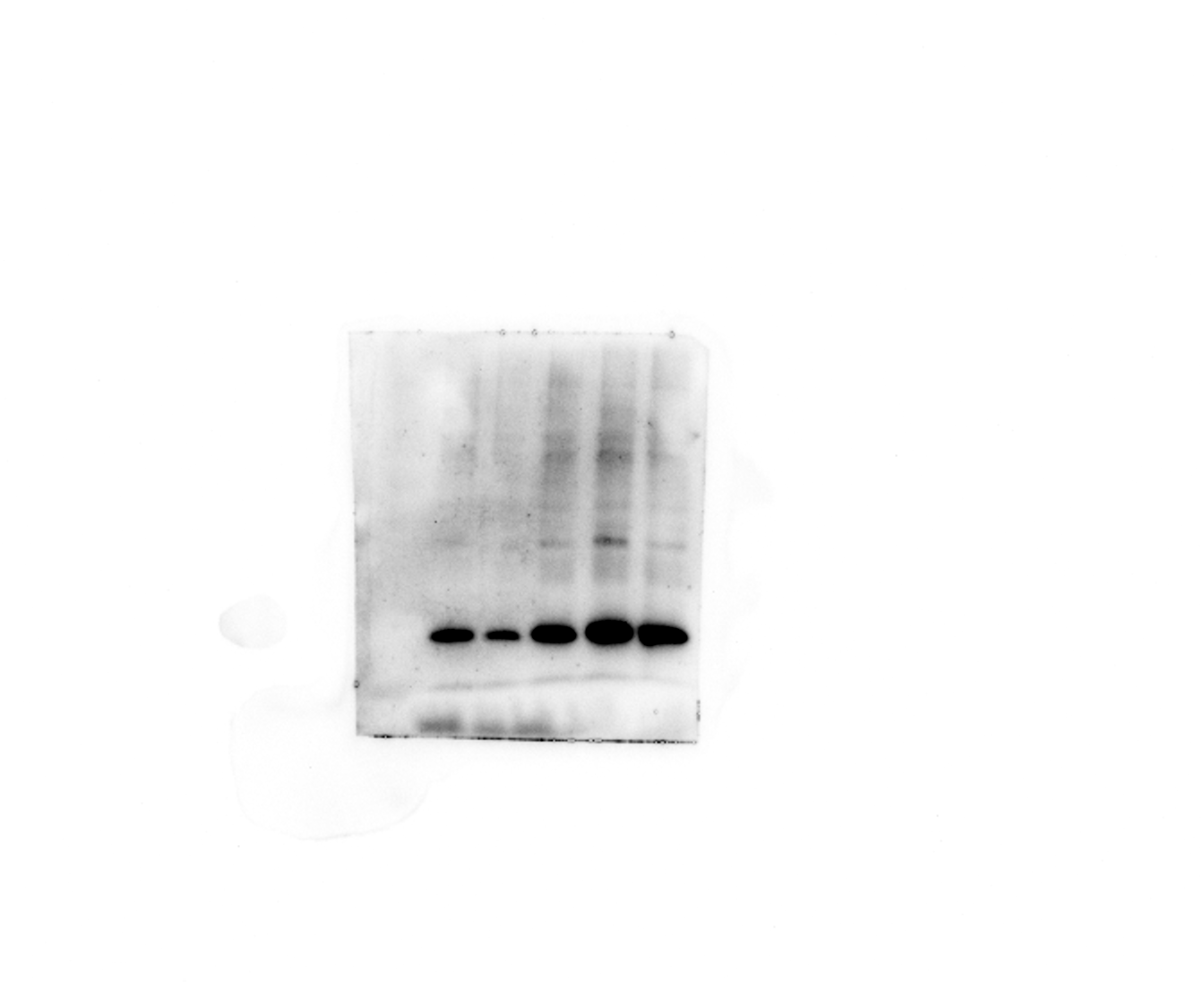


Fig S3 original gel of Bcl2


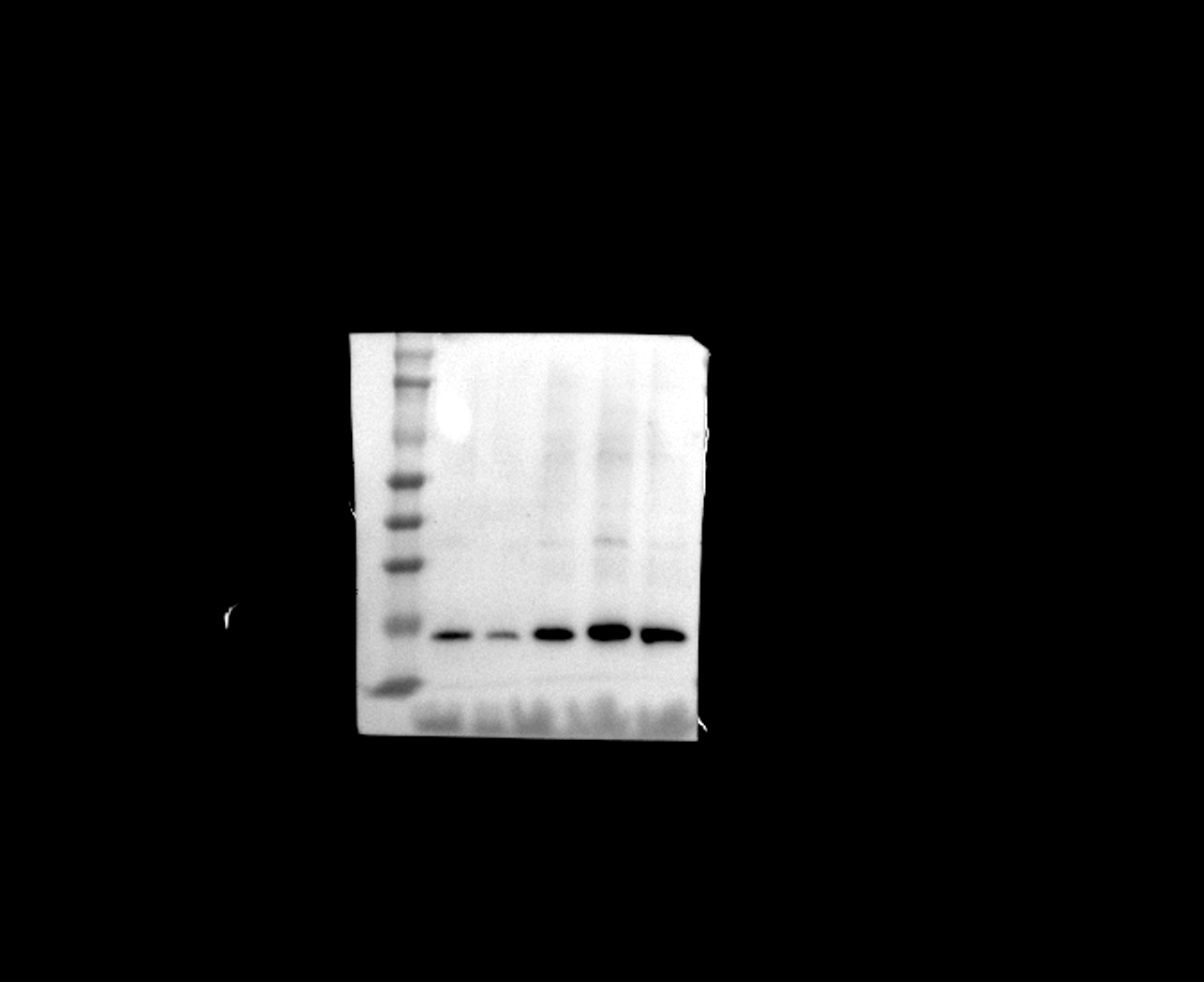


Fig S4 Bcl2-merged images of the visible light image and chemiluminescence blot


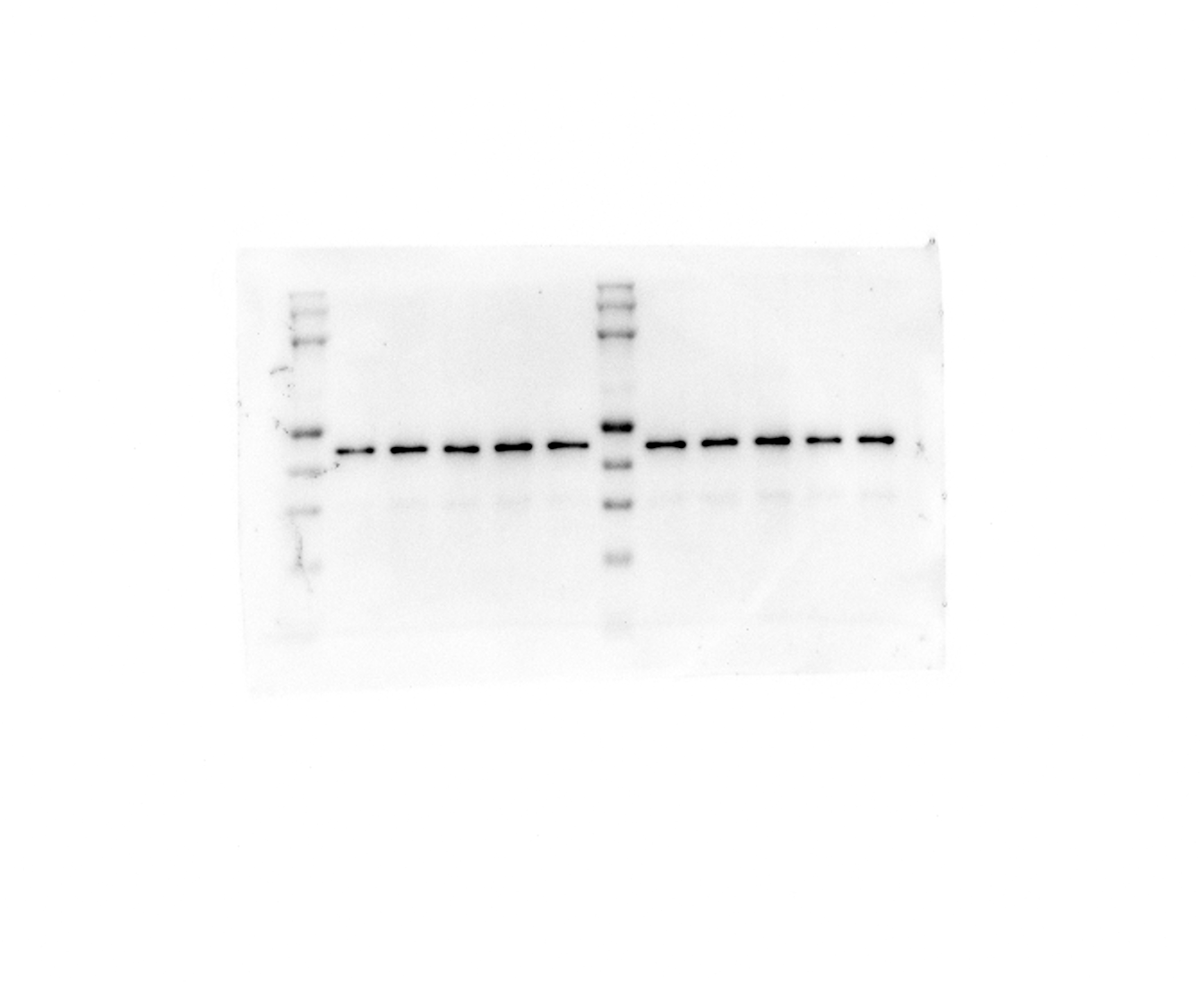


Fig S5 original gel of GAPDH


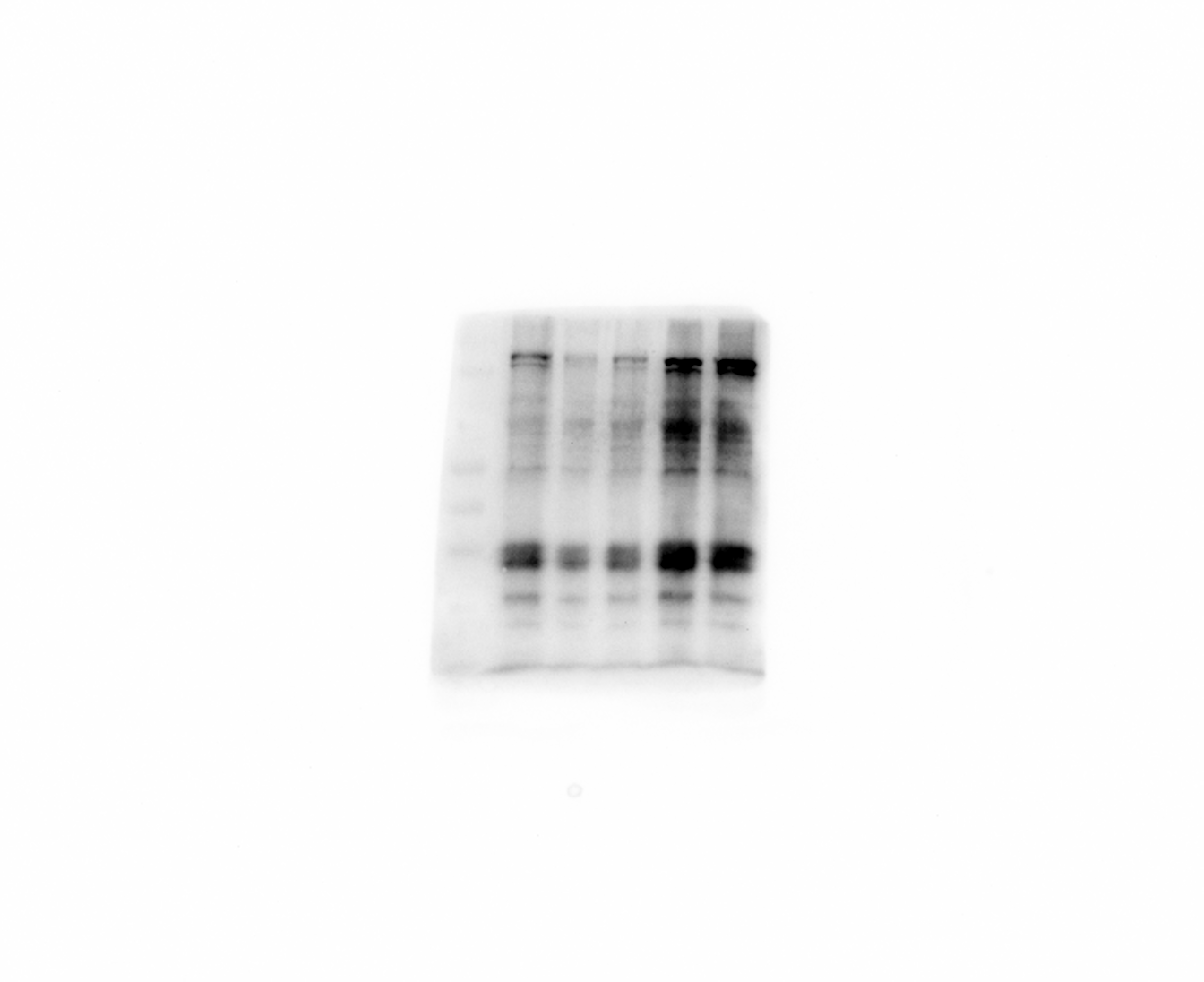


Fig S6 original gel of PI3K


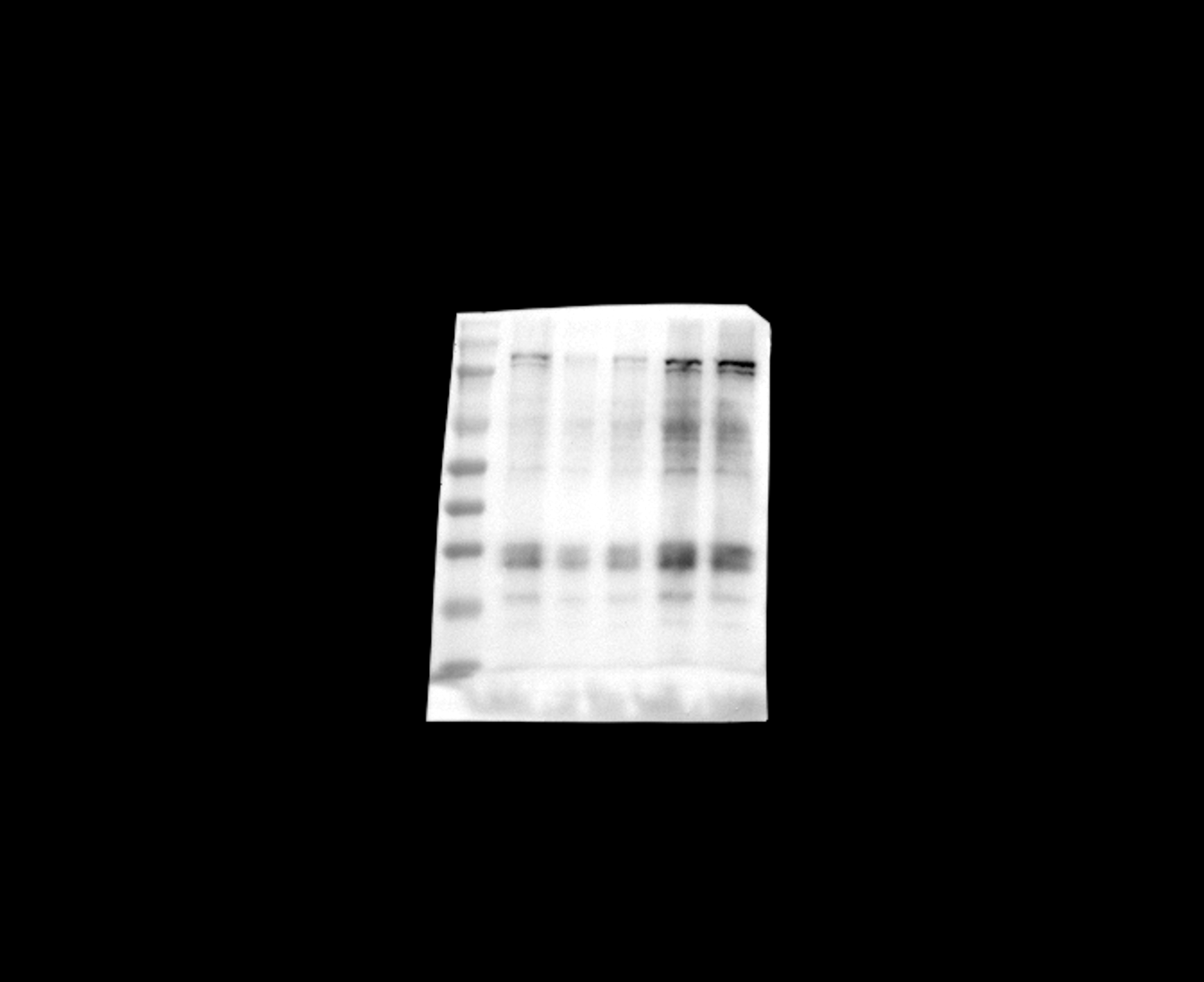


Fig S7 PI3K-merged images of the visible light image and chemiluminescence blot


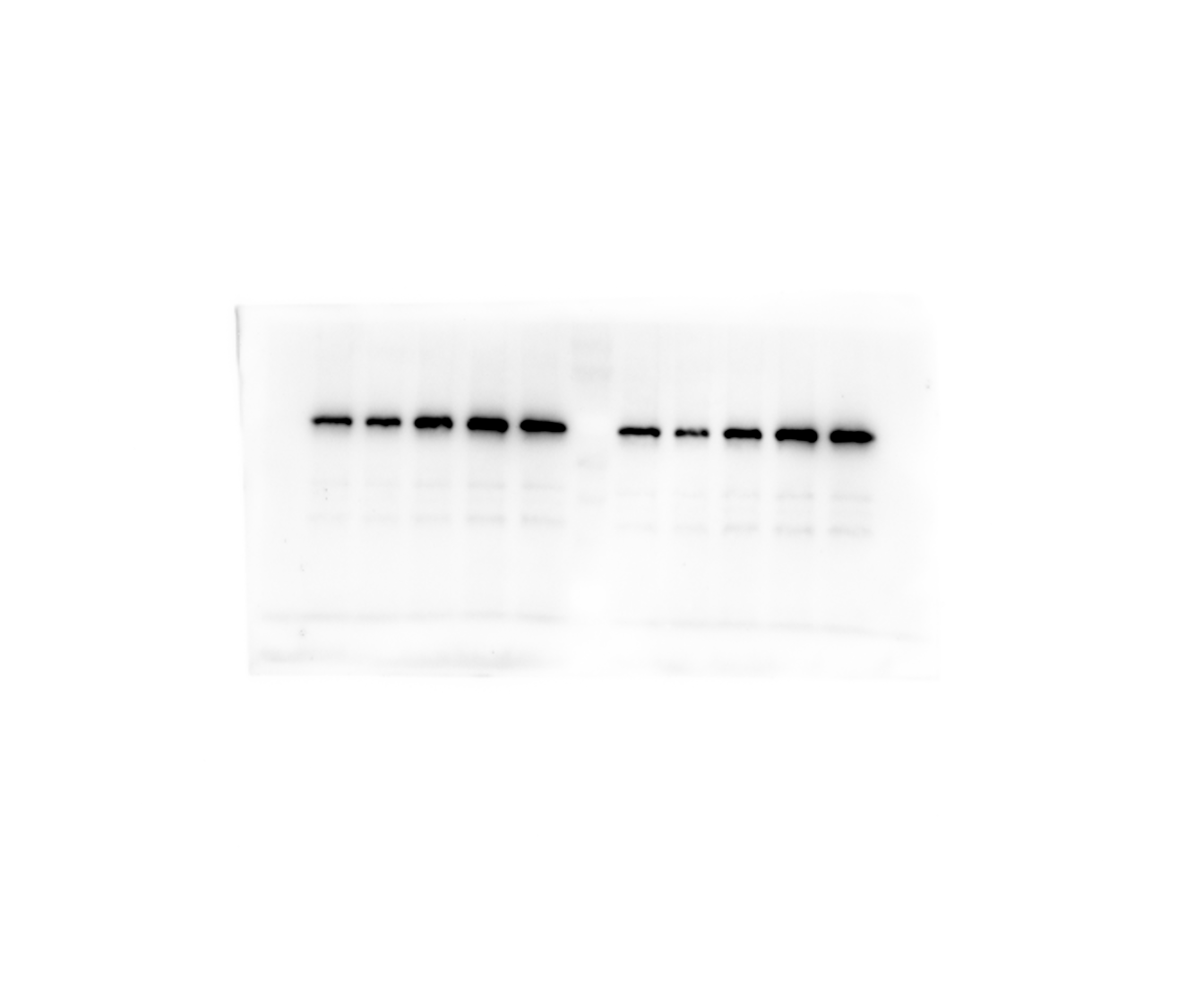


Fig S8 original gel of AKT


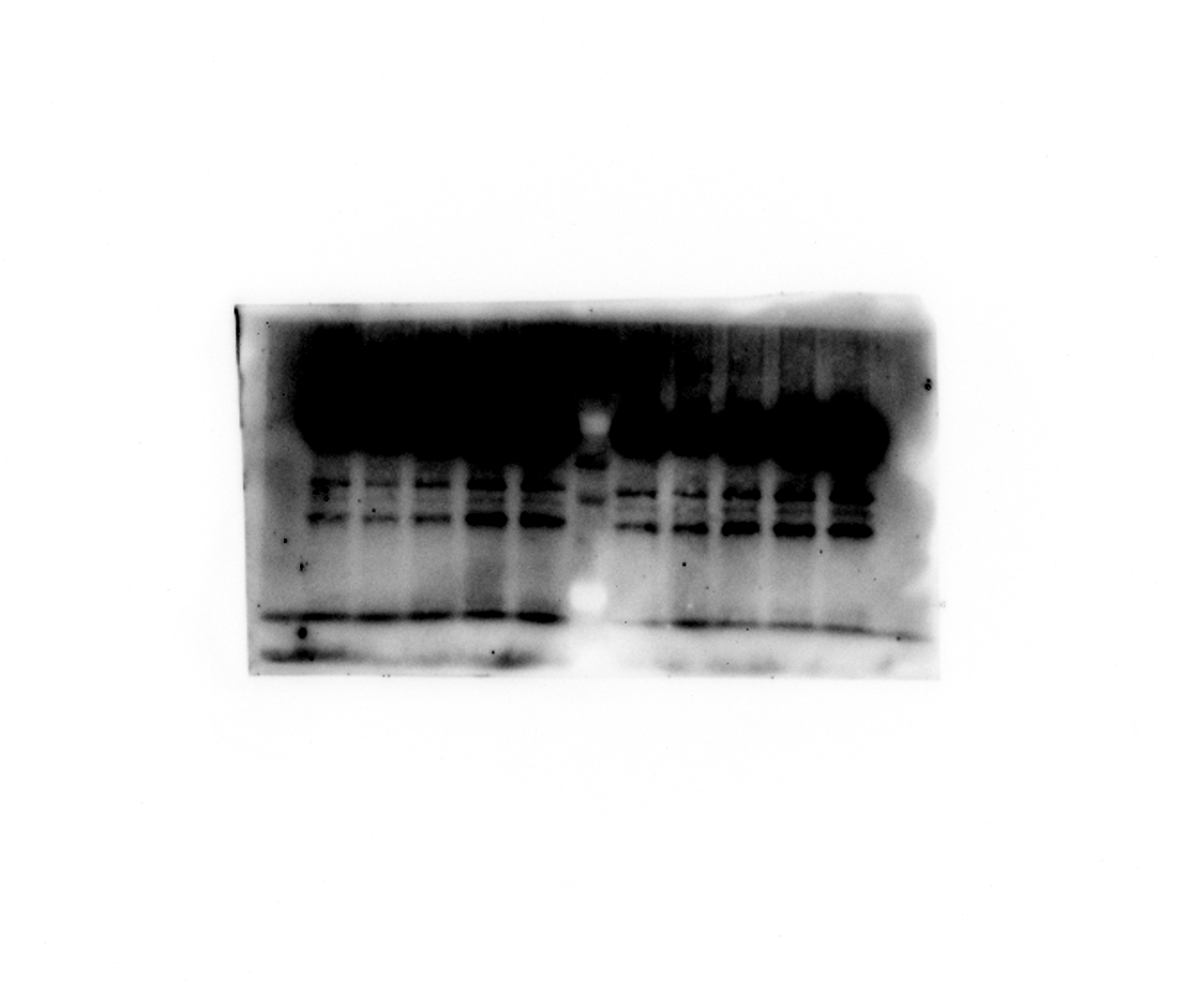


Fig S9 original gel of p-AKT


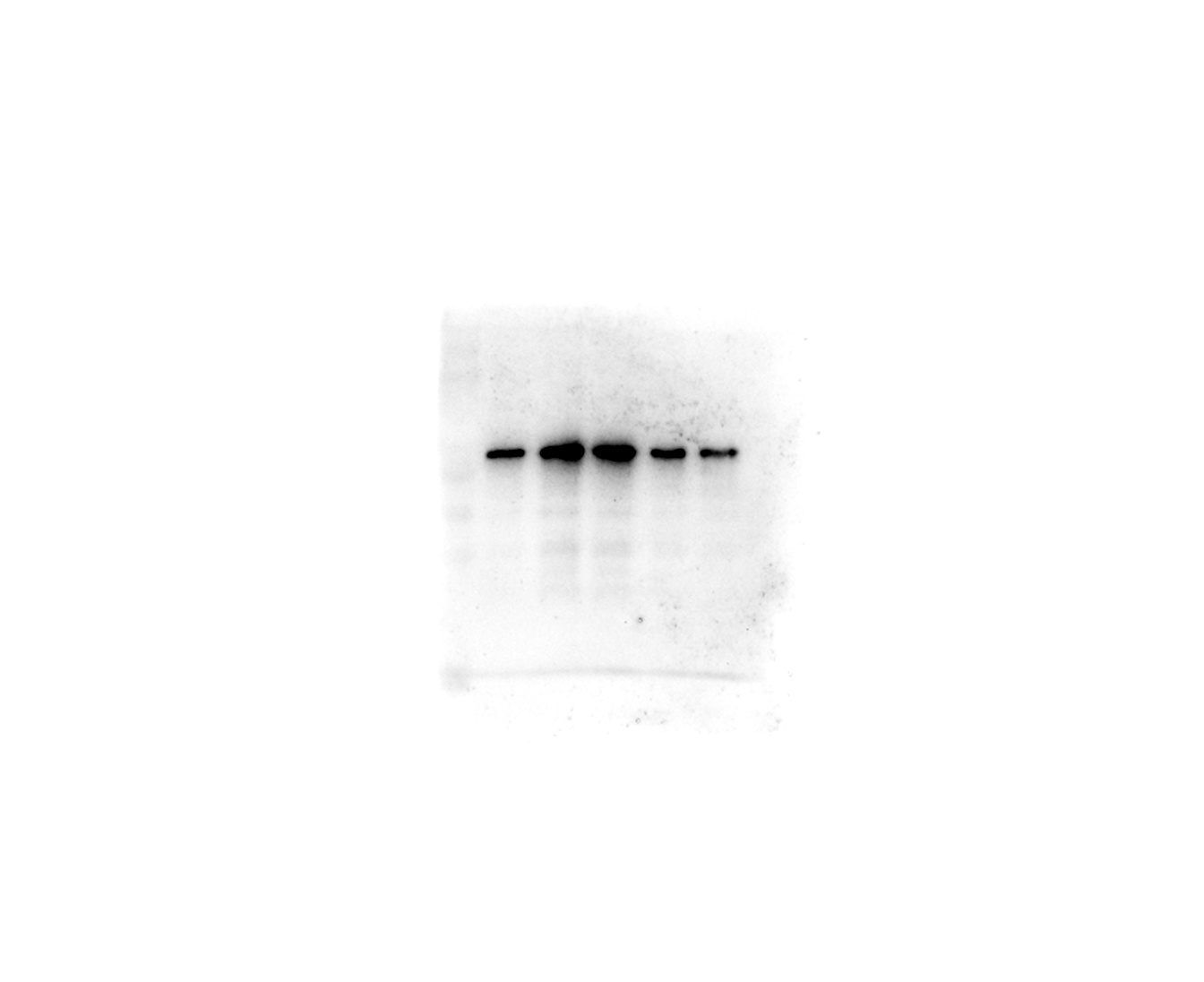


Fig S10 original gel of PTEN


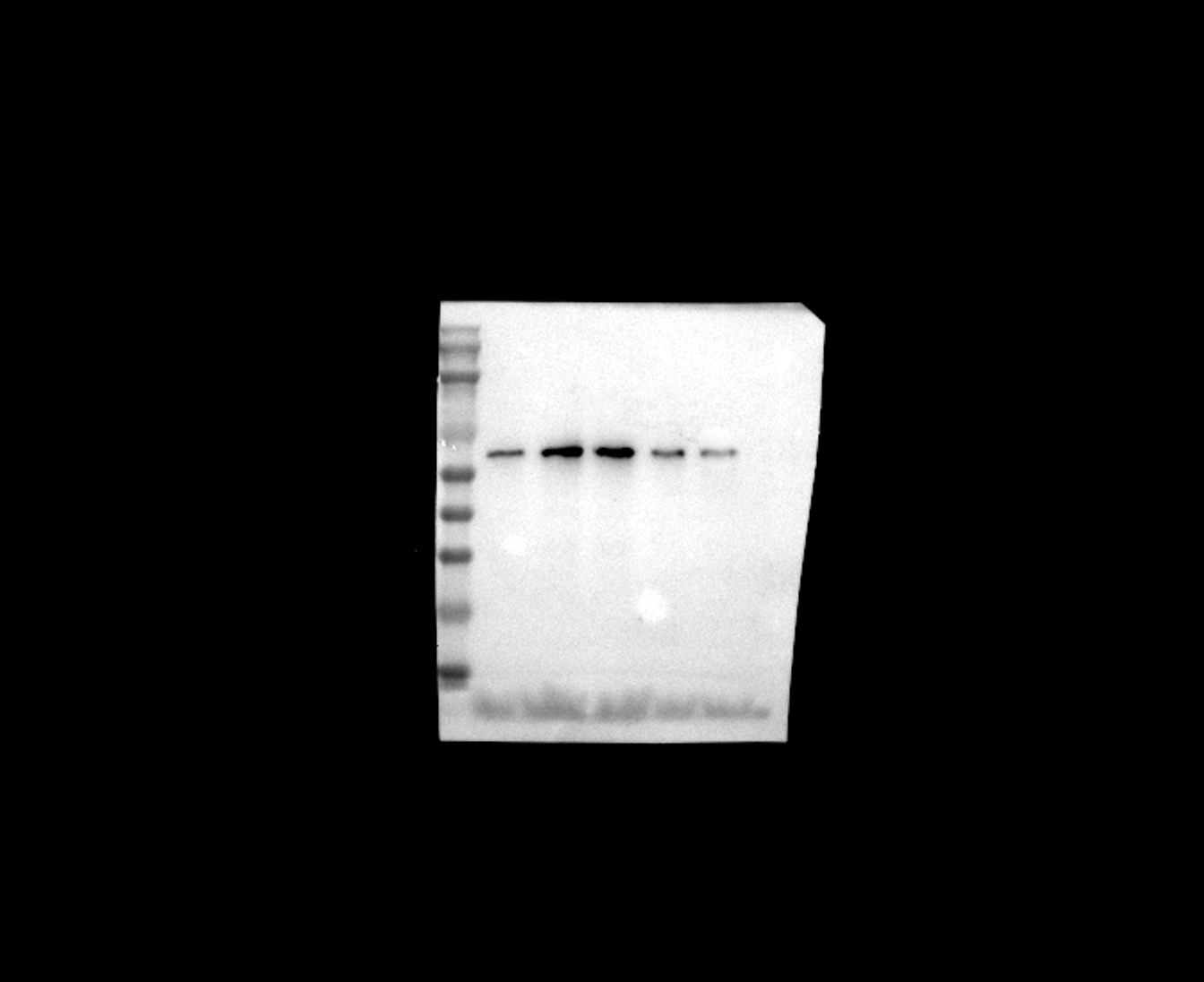


Fig S11 PTEN-merged images of the visible light image and chemiluminescence blot


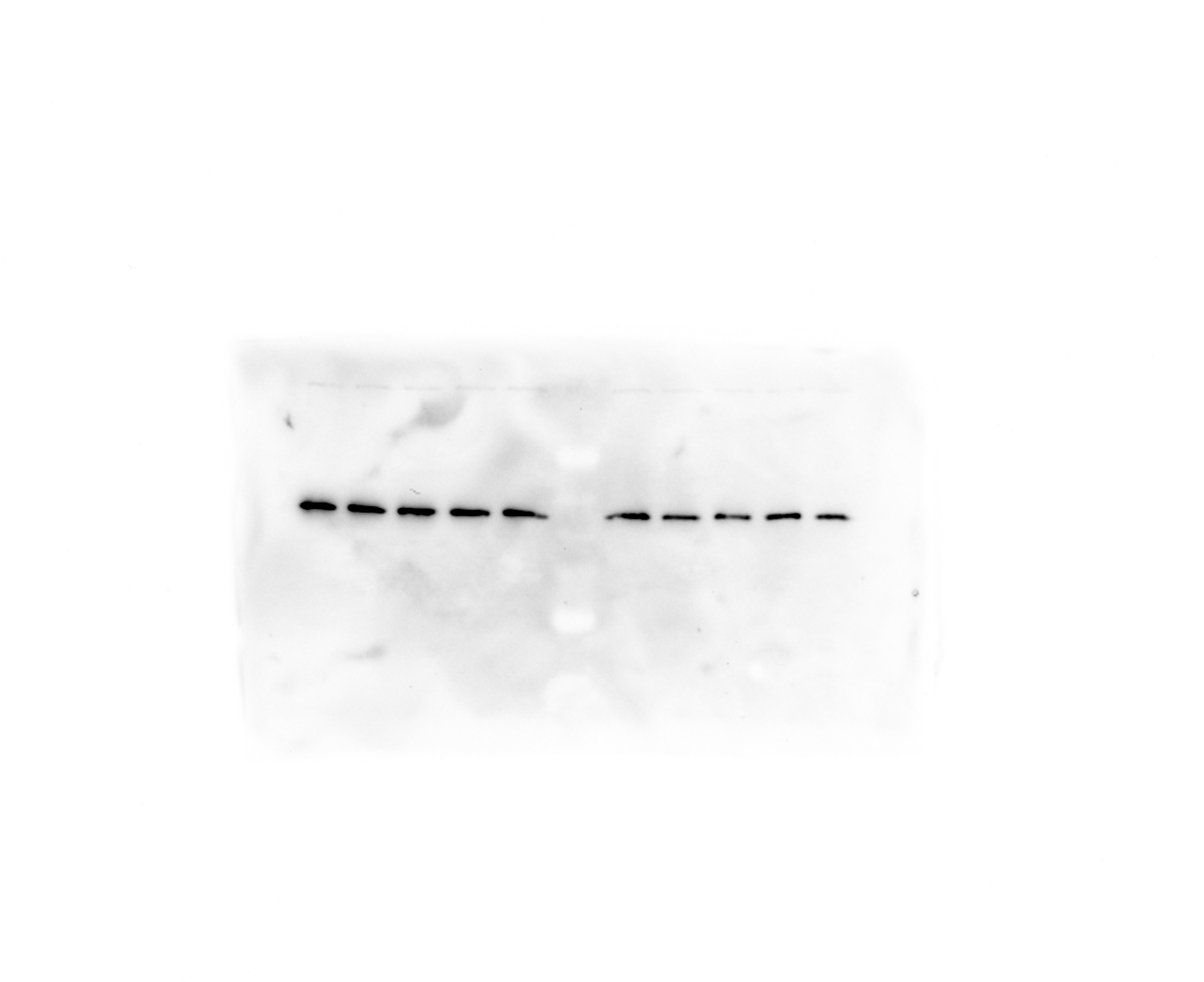


Fig S12 original gel of GAPDH
